# EEG activity represents the correctness of perceptual decisions trial-by-trial

**DOI:** 10.3389/fnbeh.2014.00105

**Published:** 2014-03-28

**Authors:** Jose L. Pardo-Vazquez, Isabel Padrón, José Fernández-Rey, Carlos Acuña

**Affiliations:** ^1^Departamento de Fisiología, Facultad de Medicina and Complejo Hospitalario Universitario (CHUS), Universidad de Santiago de CompostelaSantiago de Compostela, Spain; ^2^Departamento de Psicología Social, Básica y Metodología, Facultad de Psicología, Universidad de Santiago de CompostelaSantiago de Compostela, Spain

**Keywords:** performance-monitoring, perceptual decision-making, event-related potential (ERP), feedback, trial-by-trial

## Abstract

Performance monitoring is an executive function, which we depend on for detecting and evaluating the consequences of our behavior. Although event related potentials (ERPs) have revealed the existence of differences after correct and incorrect decisions, it is not known whether there is a trial-by-trial representation of the accuracy of the decision. We recorded the electroencephalographic activity (EEG) while participants performed a perceptual discrimination task, with two levels of difficulty, in which they received immediate feedback. Receiver Operating Characteristic (ROC) analyses were used to reveal two components that convey trial-by-trial representations of the correctness of the decisions. Firstly, the performance monitoring-related negativity (PM-N), a negative deflection whose amplitude is higher (more negative) after incorrect trials. Secondly, the performance monitoring-related positivity (PM-P), a positive deflection whose amplitude is higher after incorrect trials. During the time periods corresponding to these components, trials can be accurately categorized as correct or incorrect by looking at the EEG activity; this categorization is more accurate when based on the PM-P. We further show that the difficulty of the discrimination task has a different effect on each component: after easy trials the latency of the PM-N is shorter and the amplitude of the PM-P is higher than after difficult trials. Consistent with previous interpretations of performance-related ERPs, these results suggest a functional differentiation between these components. The PM-N could be related to an automatic error detection system, responsible for fast behavioral corrections of ongoing actions, while the PM-P could reflect the difference between expected and actual outcomes and be related to long-term changes in the decision process.

## Introduction

Efficient decision-making for adaptive behavior requires monitoring of performance in order to detect and correct errors and to adjust future behavior accordingly. Previous electrophysiological recordings in human participants have allowed the identification of two ERP components that have been interpreted as brain correlates of performance monitoring. The first one, called error-related negativity (ERN; Gehring et al., 1993) or error negativity (Ne; Falkenstein et al., 1991), is a negative deflection, with a fronto-central scalp distribution, which peaks about 80–100 ms after an incorrect response. This component is often followed by a positive deflection, called error positivity (Pe; Falkenstein et al., 1991, 1995), with a centro-parietal distribution, which peaks about 200–450 ms after an incorrect response. A negative deflection has also been found after feedback informing the participants about their performance; it has a fronto-central distribution, peaks about 250–350 ms after error feedback and is called feedback-related negativity (FRN; Miltner et al., 1997; Gehring and Willoughby, 2002; Holroyd and Coles, 2002; Nieuwenhuis et al., 2004). Regarding the positive component, when participants receive feedback after their behavioral response the results are more inconsistent. Some works describe a positive wave, which peaks about 300–600 ms after feedback, whose magnitude is higher after positive (gains) than after negative (losses) feedback and that has been interpreted as a type of P300 (Holroyd and Coles, 2002; Yeung and Sanfey, 2004; Hajcak et al., 2005; Yeung et al., 2005; Holroyd et al., 2006; Bellebaum et al., 2010; Zhou et al., 2010). In other cases, however, the positive deflection is higher after negative feedback, resembling the Pe (Frank et al., 2005; Crowley et al., 2009; San Martin et al., 2010; Schuermann et al., 2012).

Error detection, response conflict, emotional reaction and reinforcement-learning signal have been proposed as possible meanings of these waves, but their functional role remains unclear (Gehring et al., 2012; San Martin, 2012). Moreover, despite the abundant research on this topic, the functional differences between the negative and the positive components are still under debate (Taylor et al., 2007; Gehring et al., 2012; San Martin, 2012; Riesel et al., 2013). The general consensus is that these ERP components are related to performance monitoring. However, most studies have analyzed these components by averaging the brain activity across a large number of trials. Trial averaging was used to increase signal-to-noise ratio (SNR), but this technique conveys a major disadvantage: it hides inter-trial and inter-subject response variability, preventing a precise temporal characterization of these components (Philiastides et al., 2010). In contrast, single-trial methods allow revealing the origin of response variability in the analyses of performance-related ERPs (Parra et al., 2002; Philiastides and Heekeren, 2009). If these components (ERN, FRN, and Pe) are the neural correlates of performance monitoring, it will be expected that they will covariate with the outcomes of current decisions trial-by-trial.

The ROC analysis, a methodology based on signal detection theory (SDT; Green and Swets, 1966), has proved to be useful in studying trial-by-trial covariation between single neuron activity and different components of the decision process (Romo et al., 2004; Pardo-Vazquez et al., 2008, 2009). In this work, we have used this methodology to study trial-by-trial covariation between EEG activity and the correctness of the decision in humans. This technique requires almost no data pre-processing and provides direct information about the capability of EEG data to discriminate between correct and incorrect trials after feedback presentation. Recently, similar approaches have been used to study different ERP components (Debener et al., 2005; Philiastides and Sajda, 2006; Bandt et al., 2009; Philiastides et al., 2010).

In the present study we have recorded ERPs in human participants while they performed a two-alternative forced-choice task in which they had to discriminate the length of two lines. It has been found that subjects' awareness of the outcomes affects performance-related ERPs (Nieuwenhuis et al., 2001; Hajcak et al., 2004; Endrass et al., 2007; O'Connell et al., 2007; Steinhauser and Yeung, 2010); therefore, to be sure that participants can recognize correct and incorrect decisions in every trial, we gave them feedback immediately after their responses. In previous works, a delay was usually imposed between the behavioral response and the feedback presentation in order to separate response-related from feedback-related processes and, therefore, disentangle the contribution of each of these two processes to EEG activity. However, since in most sensorimotor tasks feedback closely follows behavior, here we have studied how performance monitoring is represented in the brain when the behavioral report and the feedback are kept close. The difficulty of the discrimination was manipulated to study its effect on performance-related ERPs and thus to provide new information on the functional meaning of these components. Trial-by-trial covariation between accuracy and EEG activity was assessed with ROC analysis.

## Materials and methods

### Participants

Twelve undergraduate students (six females) between 18 and 28 years old (mean = 22.6, SD = 2.41) participated in the study. All had a normal or corrected to normal vision and reported no history of neurological disorders. Each participant provided written consent to participate in the study and received 10 € per session.

### General procedure

Participants performed the behavioral task in an isolated room (provided with a one-way mirror with the experimenter's room) and watching a computer screen binocularly at 57 cm from their eyes. A chin rest was used to avoid head movements and to maintain the distance from the screen. Stimuli presentation and participants' responses were controlled with *Superlab 4.5* (www.cedrus.com). This software was used to record the participants' responses and reaction times (RTs) for the behavioral analyses (see below).

The experiment consisted of two stages. The first stage included one experimental session of about 90 min during which the participants performed 720 trials (four blocks of 180 trials separated by short resting periods) of the length discrimination task (LDT). This stage was aimed at estimating the psychometric curves for each participant. This information was then used to select the stimuli set for the second stage. The second stage consisted of two experimental sessions of about 90 min during which participants performed the LDT while we recorded the electroencephalographic activity (EEG). Each recording session included two consecutive blocks of 288 trials separated by a short resting period. Each participant performed a minimum of 1152 trials, except for one that performed 864 trials in two recording sessions. The three sessions were conducted on consecutive days at the same hour. The Bioethics Commission of the University of Santiago de Compostela approved the experimental procedures.

### Stimuli

The stimuli were stationary bright lines subtending 0.10° in width. During the first stage three lengths were used as first stimulus (S1): 1.6°, 2°, and 2.4° and 10 lengths (five longer and five shorter than S1) as second stimulus (S2) for each S1, in steps of 0.10°. The use of 10 S2 for each S1 stimulus allowed us to compute psychometric functions reliably. These functions were then used to select the stimuli set for the second stage depending on the capability of each participant to discriminate. In the second stage, three lengths were used for the S1 and 12 for the S2 (four lengths for each S1). The three S1 were selected so that they were clearly different for the participants (using, for each participant, the differences in length—between S2 and S1—that provoked 90% correct discriminations). The four S2 for each S1 were chosen so that two of them (one longer and one shorter than S1) were difficult to discriminate (60% correct discriminations) and the other two (one longer and one shorter than S1) were easy (90% correct discriminations). Visual stimuli were created on a PC Intel E8200 at 2.13 GHz, 3.37 GB of RAM, using an ATI Radeon HD 4350, presented on a LDC monitor LG Flatron 60 Hz vertical refresh rate. Screen resolution was set to 1680 × 1050 pixels during the experiment.

### Behavioral task

**Figure 1:**
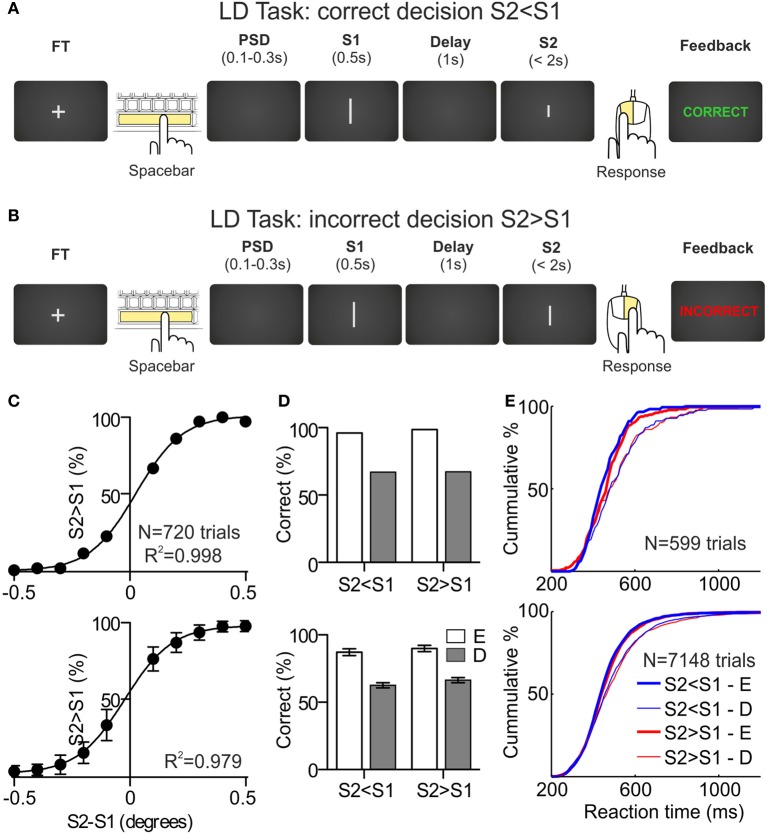
**Length discrimination task and behavioral results. (A,B)** Sequence of events during a correct and an incorrect trial, respectively, of the length discrimination (LD) task. The fixation target (FT) appears in the center of the monitor screen. The participant initiates the trial by pressing the spacebar and, after a variable pre-stimulus delay (PSD), two stimuli (white lines of variable length. S1 and S2) are presented sequentially, separated by a delay of 1 s. The first stimulus is presented for 500 ms and the second one remains on screen until the participant indicates, by pressing one of the mouse buttons, whether S2 is larger (right button) or shorter (left button) than S1. After the behavioral response, the participant receives feedback about the correctness of the choice (“Correct” or “Incorrect” in green or red, respectively). **(C)** Psychometric functions obtained during the first behavioral session for one example participant (upper panel) and averaged across the sample (*N* = 12; lower panel). **(D)** Percentage of correct responses, as a function of the sign of the difference between S2 and S1 (S2 < S1 and S2 > S1) and the difficulty of the discriminations, for one example participant (upper panel) and averaged across the sample (*N* = 12; lower panel); E, easy, D, difficult. **(E)** Cumulative distributions of the reaction times, as a function of the sign of the difference between S2 and S1 and the difficulty of the discriminations, for one example participant (upper panel) and for the sample (*N* = 12; lower panel).

Length Discrimination Task (LDT). Participants had to compare the length of two lines and decide whether the second line (S2) was shorter or longer than the first line (S1) (Figures 1A,B). Each trial began with a fixation target (FT) presented in the center of the screen until participants pressed the spacebar, then the FT disappeared and, after a variable pre-stimulus delay (PSD, 100–300 ms), two stimuli (S1 and S2) were presented in sequence, with a fixed inter-stimulus interval (ISI or delay, 1s). First stimulus was presented during 500 ms and second stimulus remained on screen until the participants indicated their decisions by pressing the right mouse button when they considered that S2 > S1 and to the left mouse button when they considered that S2 < S1; participants had 2000 ms to respond, otherwise the trial was aborted (and a text message on screen indicated to the participants that they had to respond faster). Immediately after the behavioral response, participants received information about their performance. Feedback consisted of a text message presented in the center of the screen with the word “CORRECT” (written in green) or “INCORRECT” (written in red), after correct and incorrect responses, respectively (see Figures 1A,B).

### EEG data acquisition

The electroencephalogram (EEG) was recorded from 62 scalp sites using Ag/AgCl electrodes embedded in an elastic cap (*Easycap*, Bionic Electrics Inc.) according to the international 10–20 system (Supplementary Figure 1). We used a monopolar configuration with 60 active electrodes, 2 inactive electrodes (located on the earlobes) that were used as references and a ground electrode placed between Fpz and Fz positions. Vertical eye movements (VEOG) were recorded simultaneously with the EEG using two electrodes placed on the muscles below and above the left eye orbital. Electrode impedances were kept below 10 kΩ. EEG and VEOG data were continuously recorded using *Brain Vision QuickAm-72* amplifier (www.brainproducts.com) with a band pass filter of 0.1–30 Hz and digitalized at 500 Hz for offline analyses. Data were referenced offline to the two inactive electrodes.

### Data analysis

#### Behavioral data

Data obtained for each participant in the first stage was represented as a psychometric function. It represents the probability of correct responses against the values of the stimulus variable that is being studied. In this case, the stimulus variable is the difference in length between S1 and S2. Data were fitted to a logistic Bolztmann equation:

$$p_{b}(a)=\frac{A_{1}-A_{2}}{1+e^{-\left(\frac{a-a_{0}}{a_{1}}\right)}}+A_{2}$$

where *p*_*b*_(*a*) represents the probability of a “longer than” judgment (S2 > S1); *A*_1_ and *A*_2_ represent, respectively the maximum and minimum values of *p*_*b*_(*a*); *a*_0_ is the value of size of S2 for which *p*_*b*_(*a*) = (A_1_ − A_2_)/2 and *a*_1_ represents the width of the function. These adjustments were performed using *Graph Pad Prism 5.0* (www.graphpad.com/prism), which provided statistical information about the goodness of fit. With this function, average probability values for “longer than” responses were obtained, for each value of S2. This allowed us to include, in the second stage, easy and difficult trials by choosing those S2 that provoked 60 and 90% correct responses. This selection was performed for S2 < S1 and S2 > S1 comparisons separately.

Behavioral data from the second stage (two recording sessions) was analyzed to assess the adequacy of stimuli selection and the effect of task difficulty in participants' performance both in terms of speed (RTs) and accuracy (percentage of correct judgments).

#### Event related potential analysis

Application of filters, ocular correction artifacts, segmentation and EEG data exportation were performed using *Brain Vision Analyzer 2.0*. Feedback-locked epochs of 900 ms were extracted from continuous data beginning 100 ms prior to feedback onset for all conditions: correct trials, incorrect trials, and correct and incorrect trials for easy and difficult discriminations. Correction for ocular artifacts was applied using the standard methods (Gratton et al., 1983). In order to perform trial-by-trial analyses, individual segments were exported for each subject and channel. Baseline correction (−100 to 0 ms) was applied and trials whose voltages were lower than −50 μV or higher than 50 μ V were eliminated.

ROC analyses were conducted to compare the ERPs recorded in two experimental conditions (e.g., correct vs. incorrect trials). This analytical method, based on Signal Detection Theory (SDT, Green and Swets, 1966), has been successfully used to reveal the complex relations between single-neuron activity and different components of the decision process (Romo et al., 2004; Pardo-Vazquez et al., 2008, 2009). It allows the experimenter to estimate the degree of overlap between two response distributions. Each point of the ROC curve represents the proportion of hits (*p*Hits, i.e., the proportion of trials of one condition—e.g., incorrect trials—in which the voltage reached or surpassed a criterion) against the proportion of false alarms (*p*FAs, i.e., the proportion of trials of the other condition—e.g., correct trials—whose voltage reached or surpassed the same criterion). The criterion varied between the minimum and maximum values of both distributions and a total of 100 criteria were used. As a result of applying these criteria, we constructed a curve and estimated the area under it (area under the ROC curve, AUC ROC; see Supplementary Figure 2). An AUC ROC of 0.5 means that the two distributions are completely overlapped. AUC ROC values above 0.5 indicate higher voltages after incorrect trials (i.e., *p*Hits > *p*FAs) and AUC ROC values below 0.5 indicate higher voltages after correct trials (i.e., *p*FAs < *p*Hits). An AUC ROC of 0 or 1 means that the two distributions are completely separated. The analysis was applied to every recorded time point, beginning 100 ms before the feedback onset and ending 800 ms after the response. To establish when the AUC ROC value significantly deviates from 0.5, we performed a permutation test, also as a function of time. The trials were randomly assigned to the two conditions being compared and AUC ROC values for each permutation (*n* = 200) were estimated. Minimum and maximum ROC intervals were computed as the values of the AUC ROC histogram for which the probability of finding random smaller and greater values, respectively, was less than 0.01. This stage produces temporal AUC ROC margins that change as the voltage does. These margins were approximately in the range of 0.4 and 0.6 (Wallis and Miller, 2003). The criterion for ROC significance was defined as the point at which the ROC index exceeded the limits obtained with the permutation test. We used this methodology to compare the voltages recorded after positive and negative feedback at each electrode and, therefore, to determine whether the ERPs covary with the outcomes of current decisions trial-by-trial. Similar comparisons (between correct and incorrect trials) were also performed for easy and difficult trials separately. These analyses were performed using custom-made programs in Matlab R2009b (www.mathworks.com).

## Results

### Behavioral results

#### Selection of stimuli sets

The psychometric functions for one participant (upper panel) and for the sample (lower panel; *N* = 12; Mean ± *SD*) are shown in Figure 1C. The fitting of every psychometric function was very good (*R*^2^ = 0.99). The mean differences between S2 and S1 used during the recording sessions were: (1) for S2 < S1 trials, 0.27° (SD = 0.09°) and 0.06° (SD = 0.04°) for easy and difficult discriminations, respectively, and (2) for S2 > S1 trials, 0.23° (SD = 0.1°) and 0.02° (SD = 0.04°) for easy and difficult discriminations, respectively.

#### Performance during the recording sessions

The behavioral performance of the participants was clearly different as a function of the difficulty of the discriminations, both in terms of accuracy and speed. In Figure 1D we have represented the percentage of correct decisions as a function of the difficulty of the discriminations (easy and difficult) and of the sign of the difference between S2 and S1 [sign(S2-S1); S2 < S1 and S2 > S1] for one participant (upper panel) and for the sample (lower panel; *N* = 12; Mean ± SD). For the S2 < S1 trials, the mean percentages of correct decisions for easy and difficult discriminations were 87.2 (*SD* = 8.7) and 62.6 (*SD* = 6.6), respectively; for the S2 > S1 trials, the mean percentages of correct decisions for easy and difficult discriminations were 89.9 (*SD* = 8.1) and 66.4 (*SD* = 6.8), respectively. These differences were significant (*p* < 0.001, *t*-test for dependent samples) and no significant differences were found as a function of sign(S2-S1). Note that these percentages are very close to the 90 and 60% that we used to select the stimuli sets for each participant.

The same pattern was found for the RTs obtained in correct trials: for the S2 < S1 trials, the mean RTs for easy and difficult discriminations were 464 ms (*SD* = 64 ms) and 490 ms (*SD* = 76 ms), respectively; for the S2 > S1 trials, the mean RTs for easy and difficult discriminations were 473 ms (*SD* = 59 ms) and 501 ms (*SD* = 79 ms), respectively. Both differences were significant (*p* < 0.01, *t*-test for dependent samples) and no significant differences were found as a function of sign(S2-S1). The cumulative distributions of the RTs as a function of difficulty and sign(S2-S1), for one participant (upper panel) and for the sample (lower panel; *N* = 12), are represented in Figure 1E.

### ERP results

#### Average voltage and grand mean voltage

**Figure 2:**
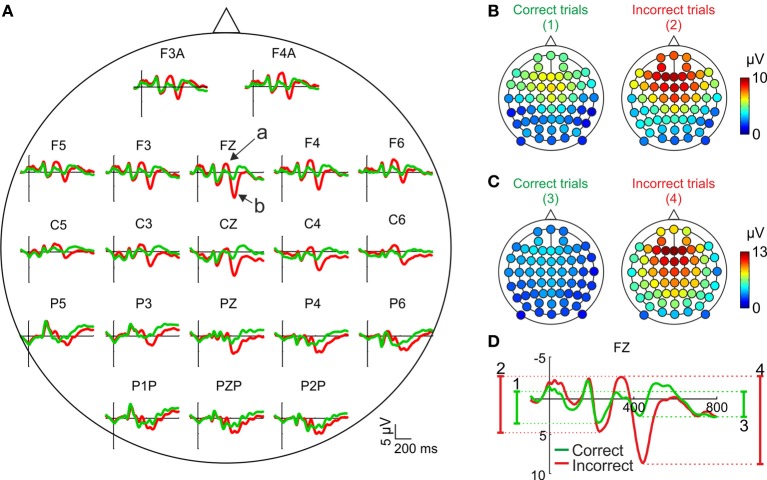
**Scalp distribution of the EEG activity after feedback presentation. (A)** Grand mean, averaged across the sample (*N* = 12), for correct (green traces) and incorrect (red traces) trials in the principal electrode positions. Time (y-axis) is from −100 to 800 ms with respect to feedback onset (signaled by the x-axis). Arrows labeled “a” and “b” signal the negative and positive deflections, respectively. Negative voltages are plotted upwards by convention. **(B,C)** Scalp distributions of the amplitudes of the negative and positive deflections, respectively, averaged across the sample (*N* = 12) after correct (left panels) and incorrect trials (right panels); color code, from blue to red, indicates the mean voltage. **(D)** Graphical explanation of the method followed to calculate these amplitudes; numbers, from 1 to 4, correspond to each of the panels in **(A,B)**.

Most studies on the relationship between performance monitoring and EEG activity are based on the comparison of the grand mean ERPs, averaged across subjects, after correct and incorrect trials. In Figure 2A we have represented the feedback-locked grand mean ERPs averaged across the 12 participants, as a function of the outcome of the current trial, for the principal electrode positions. This representation shows two time periods during which the ERPs are different as a function of the correctness of the trial. During the first period (about 300–400 ms after feedback onset) there is a negative deflection whose amplitude is higher (i.e., more negative) after negative than after positive feedback (see Figure 2A, electrode Fz, arrow a). During the second period (about 400–500 ms after feedback onset) there is a positive deflection whose amplitude is higher (i.e., more positive) after negative feedback (see Figure 2A, electrode Fz, arrow b).

The amplitude of the negative deflection was computed as the difference between the most negative peak following feedback onset in a 300–400 ms time window and the preceding positive peak between 200 and 300 ms [see Figure 2D; this method is similar to that used in previous works, e.g., (Schuermann et al., 2012)]. The amplitude of the positive deflection was computed as the difference between the most positive peak in a 400–500 ms time window and the preceding negative peak between 300 and 400 ms (see Figure 2D). The amplitude of both components is higher after incorrect trials and show a clear fronto-central distribution, although the negative deflection (Figure 2B) is slightly more anterior than the positive (Figure 2C).

Although this methodology has been successful in revealing the existence of error-related (or performance-related) differences in ERPs, it does not allow us to study whether these components covary with the outcomes of the current decisions on a trial-by-trial basis. This is a fundamental issue as most theories on the functional meaning of ERN (or FRN) and Pe are based on the assumption that these components reflect performance-monitoring processes that are only useful if they provide a reliable index of the outcomes of current behavior. Therefore, if these components are the neural correlates of performance monitoring (irrespective of the exact role they play in this executive process) it is expected that they will differentiate correct from incorrect decisions trial-by-trial, i.e., there will be a significant covariation between the voltage recorded and the accuracy of decisions.

#### Trial-by-trial covariation between ERPs and correctness of current decisions

**Figure 3:**
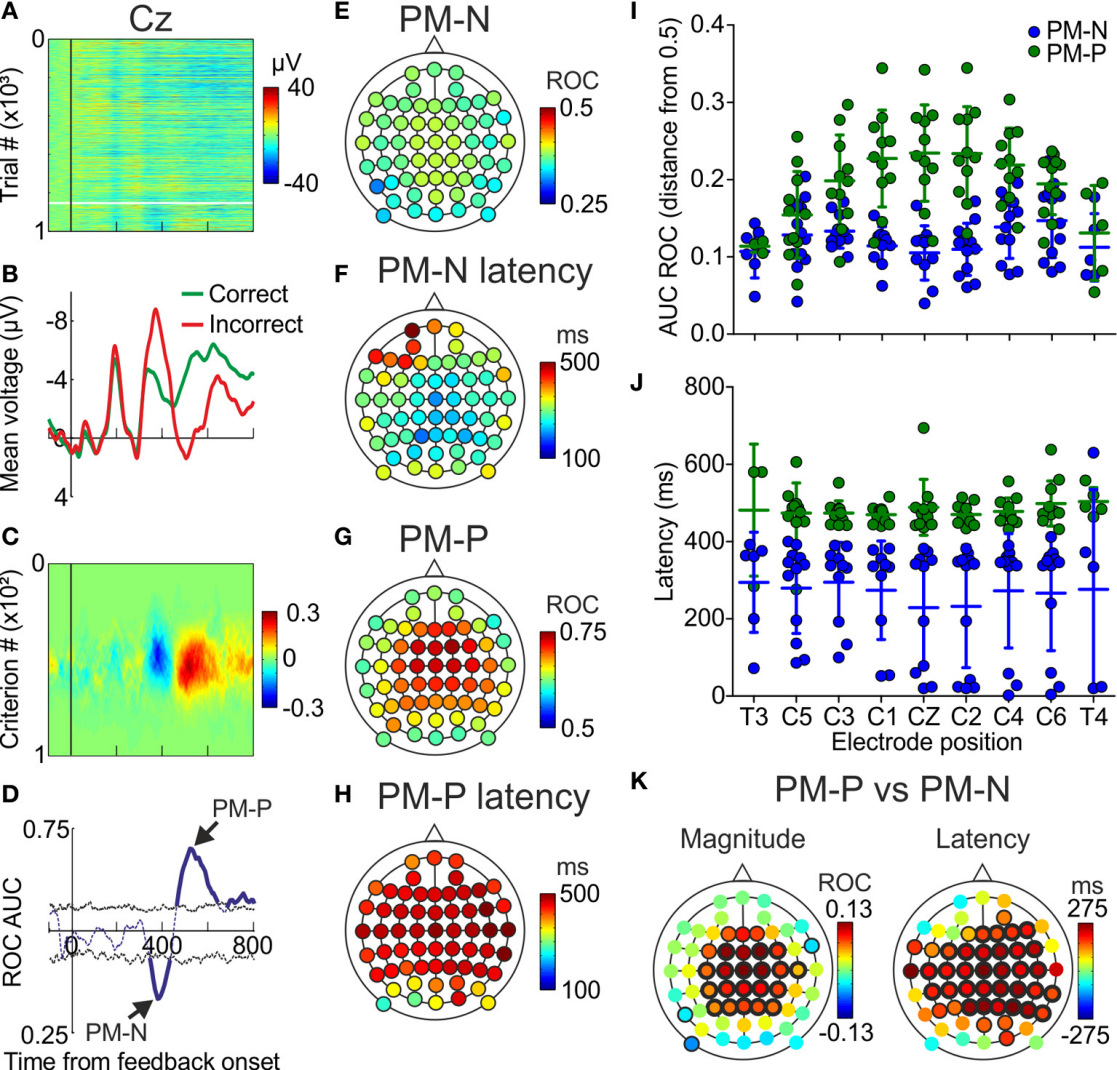
**Trial-by-trial results. (A)** Voltage, as a function of time, recorded in the electrode located at CZ of one example participant; each line represents a trial and the color code, from blue to red, the voltage; vertical black line indicates the presentation of the feedback; horizontal white line separates correct (upper) from incorrect (lower) trials. **(B)** Voltage averaged across correct and incorrect trials, as a function of time, in the same electrode location. **(C)** Difference between *p*FAs and *p*Hits, as a function of time and criterion, calculated from data in **(A)**. **(D)** AUC ROC as a function of time, for the same electrode. **(E,G)** Minima (PM-N) and maxima (PM-P) values of the AUC ROC comparing correct vs. incorrect trials averaged across the sample (*N* = 12 participants). Color code, from blue to red, indicates the AUC ROC values. **(F,H)** Latencies at minimum and maximum of the AUC ROC averaged across the sample (*N* = 12). Color code, from blue to red, indicates latency in ms. **(I)** Comparison of the capability to discriminate (distance from 0.5 in the AUC ROC values) of the PM-N and the PM-P, for 9 example electrodes; dots represent individual data; horizontal lines, and error bars, Mean ± *SD* (*N* = 12 participants). **(J)** Comparison of the latencies at the PM-N and the PM-P for 9 example electrodes; dots represent individual data; horizontal lines and error bars, Mean ± *SD* (*N* = 12 participants). **(K)** Scalp distribution of the differences between PM-N and PM-P in their capability to discriminate correct from incorrect decisions, averaged across the sample (*N* = 12) and scalp distribution of the differences in the latencies at the PM-N and the PM-P, averaged across the sample (*N* = 12); black halos indicate electrode positions where the differences were significant (thin and thick halos for *p* < 0.05 and *p* < 0.01, respectively).

Trial-by-trial analyses were conducted to compare the voltages obtained after correct (positive feedback) and incorrect (negative feedback) trials. Single-trial voltages and mean voltages recorded from one participant are represented, for one example electrode, in Figures 3A,B, respectively (see also Supplementary Figure 3A). In order to apply ROC analyses, the proportion of correct trials for which the voltage surpassed different criteria (see Materials and Methods) is considered as the proportion of hits (*p*Hits, Supplementary Figure 3B) and the proportion of incorrect trials that surpassed the same criteria is considered as the proportion of false alarms (*p*FAs, Supplementary Figure 3C). These proportions, whose differences (*p*FAs-*p*Hits) are shown in Figure 3C (also Supplementary Figure 3D), were used to estimate the area under the ROC curve (AUC ROC) at each time during the first 800 ms after the feedback presentation (Supplementary Figure 3E). These single point data were then used to give an estimation of the AUC ROC as a function of time (Supplementary Figures 3D,F). The significance thresholds of the AUC ROC (upper and lower black dashed lines in Supplementary Figures 3D,F) were obtained by using a permutation test (*n* = 200 iterations).

This analysis was applied to data recorded in 12 participants using 60 EEG electrodes (see Supplementary Figure 1 for electrode positioning). For each electrode of each participant we looked for the minimum and the maximum significant values of the AUC ROC, i.e., the best discriminations between incorrect and correct trials (see Supplementary Tables 1, 2). Minima values correspond to PM-N and maxima values to PM-P (Figure 3D). We also calculated the latencies at these minima and maxima (see Supplementary Tables 3, 4). The mean of the minima AUC ROC values (PM-N) across the sample (irrespective of the electrode) was 0.28 (*SD* = 0.07). The mean of the maxima AUC ROC values (PM-P) across the sample (irrespective of the electrode) was 0.78 (*SD* = 0.06).

The scalp distribution of PM-N (Figure 3E) shows a spread distribution of values throughout the electrode positions. The mean latency averaged across the electrodes was 295 ms (*SD* = 62 ms) from feedback onset; the mean latencies at the 60 electrode positions are shown in Figure 3F. The scalp distribution of PM-P (Figure 3G) shows a fronto-central distribution with its maximum at C2A. The mean latency averaged across the electrodes was 441 ms (*SD* = 48 ms) from feedback onset; the mean latencies at the 60 electrode positions are shown in Figure 3H.

#### The PM-P discriminates correct from incorrect trials better than the PM-N

To compare the capability of PM-N and PM-P to discriminate between correct and incorrect trials, we calculated the distance from the maxima (PM-P) and minima (PM-N) AUC ROC to 0.5 and we compared those values for every electrode. We found significant differences in 27 electrode positions (Figures 3I,K; see Supplementary Table 5); in 24 out of 27 the PM-P showed a higher discrimination capability. We also compared the discrimination capability averaged across the 60 electrode positions and we found that PM-P is significantly better than PM-N in discriminating correct from incorrect trials [0.17 and 0.13, respectively; *t*_(59)_ = −5.54, *p* < 0.001]. Furthermore, we compared the latency at PM-N and PM-P for each electrode position and we found that PM-N peaks significantly earlier at most electrodes (Figures 3J,K; see Supplementary Table 6). The mean latency at PM-P (441 ms; *SD* = 48), averaged across the 60 electrode positions, was significantly [*t*_(59)_ = −13.15, *p* < 0.001] larger than the mean latency at PM-N (295 ms; *SD* = 62).

**Figure 4:**
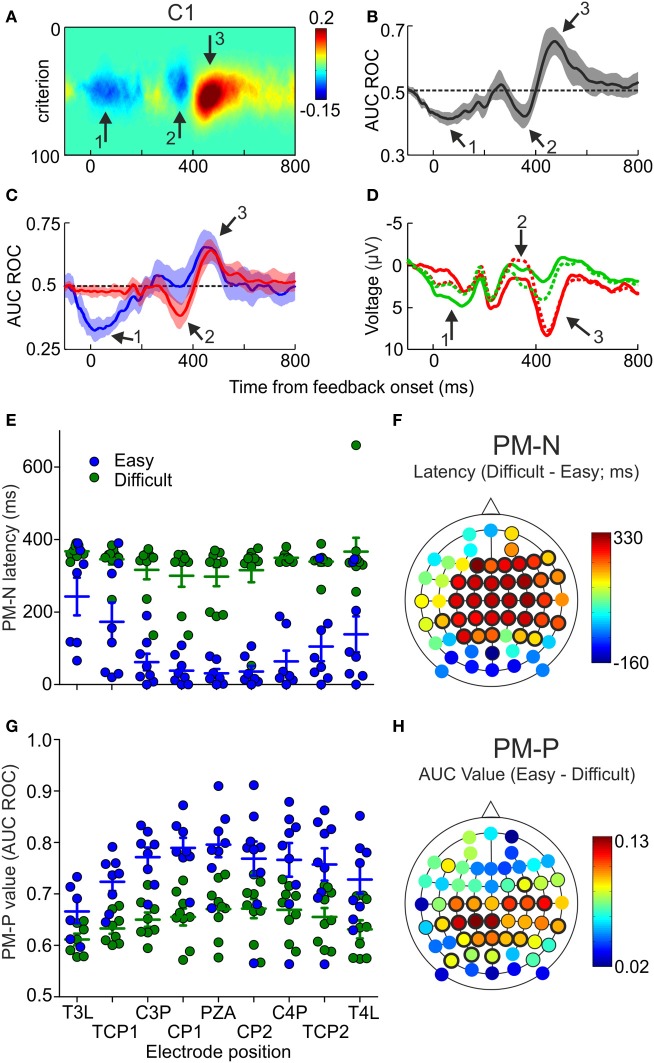
**Differences in PM-N and PM-P components as a function of the difficulty of the decisions. (A)** Difference between *p*FAs and *p*Hits averaged across the sample (*N* = 12), as a function of time and criterion, estimated at electrode C1. Arrows 1 and 2 signal the two negative and arrow 3 the positive relevant periods. **(B)** AUC ROC (*M*ean ± 2 s.e.m.), comparing the voltage recorded at electrode C1 after correct and incorrect trials, averaged across the sample (*N* = 12), as a function of time. **(C)** AUC ROC curves (*M*ean ± 2 s.e.m.), comparing the voltage recorded at different electrodes after correct and incorrect trials, averaged across the sample (*N* = 9) as a function of time and difficulty of the discriminations; blue and red lines represent the AUC ROC for easy and difficult discriminations, respectively. **(D)** Mean voltage as a function of time, recorded at electrode C1, averaged across the sample (*N* = 9), for easy correct (green solid line), easy incorrect (red solid line), difficult correct (green dashed line) and difficult incorrect (red dashed line) trials. **(E)** Comparison of the latencies at minima values of the AUC ROCs (PM-N) after easy and difficult discriminations for 9 example electrodes; dots represent individual data; horizontal lines and error bars, Mean ± *SD* (*N* = 9 participants). **(F)** Scalp distribution of the difference between the latencies at minima of the AUC ROCs (PM-N) obtained after easy and difficult comparisons, averaged across the sample (*N* = 9); black halos indicate significant locations (thin and thick halos for *p* < 0.05 and *p* < 0.01, respectively). **(G)** Comparison of the maxima values of the AUC ROCs (PM-P) after easy and difficult discriminations for 9 example electrodes; dots represent individual data; horizontal lines and error bars, Mean ± *SD* (*N* = 9 participants). **(H)** Scalp distribution of the difference between the maxima values of the AUC ROCs (PM-P) obtained after easy and difficult comparisons, averaged across the sample (*N* = 9); black halos indicate significant locations (thin and thick halos for *p* < 0.05 and *p* < 0.01).

Until now, we have focused this description in the maxima and minima values of the AUC ROC, i.e., the most reliable differences between the ERPs following correct and incorrect trials. However, it is also possible that the ERPs covariate with the outcomes during other time periods, as it seems to be the case during the first 100 ms after feedback in the electrode shown in Figure 2D. Therefore, we looked at the temporal profile of the differences between the ERPs after correct and incorrect trials, for each electrode and across the sample. For one example electrode (C1), the mean of the difference between *p*FAs and *p*Hits as a function of time and criterion and the mean of the AUC ROC as a function of time, both averaged across the sample, are represented in Figures 4A,B, respectively (see Supplementary Figures 4, 5 for more example electrodes). Looking at the sample-averaged data, two negative periods (Figures 4A,B, arrows 1 and 2) followed by a positive one (Figures 4A,B, arrow 3) are observed. These two negative periods could represent two different ERP components or, alternatively, one component that shows a different timing depending on some behavioral variable. Note that when referring to the AUC ROC we use the terms “positive” and “negative” for values that are higher and lower than 0.5, respectively.

#### Effects of task difficulty on PM-N and PM-P

Different theories on the functional role of FRN and Pe predict that the timing and/or the amplitude of these components would depend on different behavioral variables, e.g., the difficulty of the task (Pailing and Segalowitz, 2004; Krigolson and Holroyd, 2006; Holroyd and Krigolson, 2007; Masaki et al., 2007; Endrass et al., 2012). Therefore, we included two levels of task difficulty to study its influence on performance-related components. As we were interested in the information conveyed by the EEG activity in each trial, ROC analysis was conducted to study the effect of task difficulty on performance-related ERPs (i.e., PM-N and PM-P). This analysis was performed only for those participants (9 out of 12) for whom we had at least 20 trials for each condition.

We calculated two AUC ROCs as a function of time, comparing the ERPs after correct and incorrect decisions, for easy and difficult trials separately. The mean of these AUC ROCs, averaged across the sample, is shown in Figure 4C for the same electrode as in Figures 4A,B (more example electrodes are represented in Supplementary Figure 6). Visual inspection of these results shows that: (a) the first negative period (during the first 200 ms after feedback onset; see Figure 4C, arrow 1) is significant only for the easy trials; (b) the second negative period (about 350 ms after feedback onset; see Figure 4C, arrow 2) appears only after difficult trials and (c) the positive period (about 450 ms after feedback onset; see Figure 4C, arrow 3) reaches higher values after easy trials. Grand mean ERPs, averaged across the sample (*N* = 9), show the same pattern (Figure 4D). This was confirmed by comparing, for each electrode, the magnitude and the latency of PM-N and PM-P for easy and difficult trials. There were no significant differences in the magnitude of PM-N at any electrode position (Supplementary Table 7) and, at most electrode positions, the latency at maxima of PM-N was significantly lower after easy than after difficult trials (Supplementary Table 8; see also Figure 4E for nine example electrodes). Regarding PM-P, we found that the difficulty affects this component in a completely different way: the magnitude of PM-P was significantly higher for easy trials at most positions (Supplementary Table 9; see also Figure 4G for nine example electrodes) but the timing of this component was not significantly different as a function of the task difficulty (Supplementary Table 10).

The scalp distributions of the differences in latency (PM-N) and magnitude (PM-P) are represented in Figures 4F,H, respectively. The higher difference in the latency at PM-N (334 ms) was found at frontal position F1 and the higher differences in the value of PM-P (0.13) were found at central positions C3P and C1P. The mean PM-N values, averaged across the 60 electrodes, were 0.34 (*SD* = 0.02) for easy discriminations and 0.35 (*SD* = 0.02) for difficult discriminations; the mean latencies at PM-N were 191 ms (*SD* = 136 ms) for easy discriminations and 341 ms (*SD* = 37 ms) for difficult discriminations. The mean PM-P values were 0.72 (*SD* = 0.04) for easy discriminations and 0.65 (*SD* = 0.03) for difficult discriminations; the mean latencies at PM-P were 420 ms (*SD* = 50 ms) for easy discriminations and 428 ms (*SD* = 59 ms) for difficult discriminations.

#### Frequency of stimuli cannot explain PM-N and PM-P

There is evidence that the ERPs elicited by frequent and infrequent stimuli can be different (Sutton et al., 1965; Courchesne et al., 1975; Squires et al., 1975; Johnson and Donchin, 1979; Fitzgerald and Picton, 1983; Eimer, 1993; Picton et al., 2000; Holroyd et al., 2008). Therefore, because in this work incorrect trials are less frequent than correct ones, PM-N and PM-P could be related to difference in stimuli frequency. To rule out this possibility we conducted, with five participants, a control experiment in which the difficulty was adjusted so as to provoke the same proportion of correct and incorrect discriminations. The results of this control experiment were equivalent to those previously described: a negative deflection whose magnitude was higher (i.e., more negative) after incorrect trials (PM-N) followed by a positive deflection whose magnitude was higher (i.e., more positive) after incorrect trials (PM-P). Furthermore, we found a significant trial-to-trial covariation between these components and the outcomes of current decisions (Supplementary Figure 7).

## Discussion

In this work we show, using ROC analysis, evidence of a trial-by-trial covariation between ERPs and correctness of decisions in a visual discrimination task. Two ERP components represent, trial-by-trial, the outcomes of recent decisions. The first component (PM-N) peaks about 300 ms after feedback, is more negative after negative than after positive feedback and it has a spread distribution throughout the scalp. The second component (PM-P) peaks about 450 ms after feedback, is more positive after negative than after positive feedback and it is maximal at fronto-central electrode positions. The control experiment allowed us to rule out stimuli frequency as an alternative explanation for the differences in the ERPs after positive and negative feedback.

The main goal of this work was to determine whether it is possible to discriminate correct from incorrect decisions by looking at the EEG activity trial-by-trial. In order to do this, it was fundamental that trials were equivalent, except for the variable to be compared (i.e., the correctness of the choice). In sensory discrimination tasks without feedback, one of the main sources of variability across trials is the detection of errors as a result of internal monitoring processes. Correct detection of incorrect choices is more likely to happen in easy trials compared to difficult ones, but even within these categories there might be differences across trials. Therefore, we decided to give feedback immediately after the behavioral response in order to maximize the homogeneity of the participants' awareness about their performance in each trial. That is, we ensured that if the ROC analysis failed to classify one trial as correct or incorrect, it was not due to the participants' failure to identify it. Another advantage of immediate feedback is that it matches many natural situations in which the outcomes follow behavior without any delay, as it is the case for many sensorimotor tasks. However, the temporal proximity between the behavioral response and the feedback presentation imposes some limitations in the interpretation of our results. The EEG activity recorded during the analyzed epoch (from 100 ms before to 800 ms after feedback presentation) is most likely reflecting not only feedback, but also motor and response-related processes. Given that performance is symmetric (participants made approximately the same number of errors in both directions) there were no systematic differences between incorrect and correct trials with respect to the motor actions performed and, therefore, motor-related activity cannot explain the differences found in EEG activity as a function of the outcomes. The current design was aimed at analyzing how performance monitoring is represented in the EEG activity when feedback closely follows behavioral responses. It did not allow us to disentangle the contribution of response and feedback-related processes to the recorded activity, but the behavioral task can be easily adapted to use the traditional delayed feedback and address this point in future experiments.

Given that the feedback messages for correct and incorrect trials had different visual properties, it is possible that PM-N and PM-P are a reflection of this sensory difference. If this were the case, it would be expected that same feedback stimuli would evoke similar EEG responses. Our results suggest that this is not the case, since we observed significant differences in both components, as a function of the difficulty, while the visual properties of the feedback were exactly the same.

Performance-related components (i.e., ERN, FRN, and Pe) have been measured using different methods (e.g., peak-to-peak or area under the component curve). Thus, it has always been difficult to compare the results obtained in different studies (Gehring et al., 2012). Moreover, all these methods are based on averaging large number of trials and, therefore, they do not allow the experimenter to study trial-by-trial relations between EEG activity and behavior. Here we have applied a novel methodological approach, ROC analysis, which has been successful in revealing single-neuron representations of different behavioral variables on a trial-by-trial basis (Romo et al., 2004; Pardo-Vazquez et al., 2008, 2009) and that has been recently applied in studying other ERP components (Philiastides and Sajda, 2006; Bandt et al., 2009; Philiastides et al., 2010). The principal advantages of ROC analysis are that it takes into account the information conveyed by the EEG activity in each trial, it requires almost no pre-processing of the EEG data and it can be easily interpreted (Bandt et al., 2009). This approach allows the experimenter to describe performance-related components in terms of their capability to discriminate correct from incorrect outcomes and it is closely related to the functional utility of performance monitoring in decision-making: to represent the consequences of each of our choices to adjust future behavior accordingly. Previous works have addressed the relation between ERN and behavioral adjustments, with inconsistent results (Gehring et al., 2012). For example, in an experiment in which EEG and fMRI data were analyzed, Debener et al. (2005) found a systematic relation between single-trial ERN and behavior in the subsequent trial. Similar results were found in other studies (Gehring et al., 1993; Rodriguez-Fornells et al., 2002; Ladouceur et al., 2007). In other cases, however, the amplitude of this negativity showed no significant effect in post-error performance (Gehring and Fencsik, 2001; Hajcak et al., 2003; Dudschig and Jentzsch, 2009). With some modifications in the task design, our approach may shed light to our understanding of the relation between performance monitoring and behavioral adjustments. Firstly, by adding temporal structure to the sensory stimulus to be discriminated (so that integration in time will improve participants' accuracy), it would be possible to study the covariation between RTs and trial-by-trial representations of the outcomes; i.e., whether participants allocate more time for processing the sensory information after incorrect trials. Secondly, by giving the participants the possibility to change their initial choice, it will be possible to analyze the relation between EEG representations of the outcomes and choice corrections; i.e., whether an early ERP component can predict these corrections.

The timing of the PM-N that we have found is equivalent to that previously reported, for FRN, in behavioral tasks in which feedback is presented (Miltner et al., 1997; Gehring and Willoughby, 2002; Holroyd et al., 2004; Nieuwenhuis et al., 2004; Donkers et al., 2005; Bellebaum et al., 2010). Regarding its scalp distribution, although the negative deflection is higher (more negative) next to the midline, ROC analysis has shown that the higher covariation between PM-N and outcomes is observed at lateral electrode positions.

In experiments in which no feedback was presented, ERN was often followed by a positive deflection (Pe) that is higher (more positive) after error trials (Gehring et al., 1993; Falkenstein et al., 1996, 2000; Nieuwenhuis et al., 2001). This pattern of EEG activity has not been consistently found after feedback informing the participants about their performance. In some cases, a positive component (designated as P300) has been described but, unlike the Pe, it showed higher voltages after positive feedback (Hajcak et al., 2005; Holroyd et al., 2006; Zhou et al., 2010; San Martin, 2012). In other cases, however, the amplitude of the positive deflection was higher after negative feedback (Frank et al., 2005; Crowley et al., 2009; San Martin et al., 2010; Schuermann et al., 2012). In the present work we have found more positive voltage values after negative feedback, resembling the Pe component that follows ERN when no feedback is presented. Moreover, the control experiment shows that PM-P discriminates incorrect from correct trials even when they are equally frequent. Interestingly, although previous works have focused on the FRN (Falkenstein et al., 1995; Luu et al., 2000; Gehring and Willoughby, 2002; Holroyd and Coles, 2002; Holroyd et al., 2004; Hajcak et al., 2005; Nieuwenhuis et al., 2005), our results show a higher covariation between the PM-P and the accuracy of recent decisions; i.e., it has better capability to discriminate correct from incorrect trials.

Regarding the functional meaning of this positive deflection, different theories have been proposed (Falkenstein et al., 2000). It has been suggested that ERN and Pe are part of a single, oscillatory potential (Endrass et al., 2007), but there is evidence supporting that this component is functionally independent from the ERN and it is involved in additional processing of the error. Three hypotheses have been proposed to concretize the meaning of this additional processing (Falkenstein et al., 2000; Overbeek et al., 2005). Firstly, this component may be involved in the emotional evaluation of the error and its consequences. The effect of task difficulty on the PM-P is consistent with this hypothesis: the amplitude of this component is significantly higher after easy trials, in which participants are expected to have stronger emotional responses to errors. Secondly, the positivity may be related to behavioral adjustments aimed at correcting errors. As previously discussed in relation to PM-N, some features of our task make it difficult to test this hypothesis with the current design. Finally, it has been suggested that these positive components (Pe and P300) depend on the participants' awareness of the outcomes of current decisions (Nieuwenhuis et al., 2001; Hajcak et al., 2004; Endrass et al., 2007; O'Connell et al., 2007; Ridderinkhof et al., 2009; Hughes and Yeung, 2011). This is consistent with our results, as in the present work we provided feedback to the participants and, therefore, they knew the outcome of each decision. However, we have found that the trial-by-trial covariation between the voltage and the outcome of the current trial can be different, as a function of task difficulty, even when participants receive feedback informing them about their performance and, therefore, they were equally aware of the outcome of every trial. This suggests that PM-P is affected by other variables besides the participants' awareness of the outcomes.

Difficulty affects the timing of PM-N, which peaks earlier after easy trials, and the magnitude of PM-P, which is higher after easy trials. This pattern of results suggests that PM-N and PM-P represent two different processes. Firstly, PM-N could be the brain correlate of a fast error-detection system that represents the outcome of ongoing behavior as soon as possible in order to execute corrective actions within the same trial. Therefore, our results are consistent with the idea of an early negative component related to on-line error correction that has been previously suggested (Burle et al., 2008). This could explain our results regarding PM-N latency as far as errors are detected earlier after easy trials (in which the errors are more evident). Moreover, this could also explain the difference in the discrimination capability of PM-N and PM-P in our task, since participants cannot correct their decisions within the same trial, and therefore this fast-correction system is not useful. The effect of difficulty on PM-N may be the due to differential contribution of response and feedback monitoring processes; in easy trials, participants can be aware of errors even before they execute the behavioral response, while in difficult trials the detection of wrong choices may relay in feedback information and, therefore, it would be delayed with respect to the easy trials. This interpretation is consistent with the short PM-N latencies (<100 ms) observed in some electrodes after easy trials. Recently, it has been found that difficulty affects the ERN, whose amplitude is lower after difficult trials (Endrass et al., 2012; Kaczkurkin, 2013). Regarding the temporal evolution of this component as a function of task difficult, no difference has been described. Therefore, it seems that difficulty has different effects on the PM-N and the ERN, suggesting a differentiation of these components. Alternatively, task differences with previous works could explain this differentiation. Secondly, PM-P could reflect the difference between the expected and the actual outcomes because its magnitude is larger in the easy than in the difficult trials. This difference could be useful for improving the decision process in future trials. This could be accomplished by increasing the attentional resources devoted to the task after errors or by changing one or more components of the decision process (e.g., decision variable or decision rule, see (Gold and Shadlen, 2007). Consistent with our interpretation of PM-P, in previous works it has been suggested that the positive component observed after the behavioral response (Pe) could be related to post-error correction strategies (Falkenstein et al., 2000; Overbeek et al., 2005). The present work was not aimed at understanding this process and future studies will be necessary to describe the influence of performance-related ERPs on long-term changes in the decision-process. The pattern of results described in the present work is coherent with a recent proposal by Philiastides et al. (2010). These authors suggest that feedback information is processed in two sequential stages. The first one, between 180 and 380 ms after feedback onset, is described as a categorical evaluation of the valence (positive vs. negative) of the outcomes. The second one, which takes place about 300 ms after feedback, would be a quantitative representation of the valence and magnitude of the prediction error (i.e., the difference between the expected and the actual outcomes).

In this work we provide evidence on the existence of a reliable, trial-by-trial relationship between two ERP components, PM-N and PM-P, and the outcomes of current decisions. Using ROC analyses we were able to accurately categorize correct and incorrect trials by looking at the EEG activity. This gives strong evidence supporting that PM-N and PM-P represent cognitive processes related to performance monitoring. Since these components are influenced differently by the difficulty of the task in progress, they seem to reflect different cognitive processes. The PM-N could be related to an automatic error detection system because its latency is shorter after easy than after difficult trials; this system could be responsible for fast behavioral corrections of ongoing actions. The PM-P could reflect the difference between expected and actual outcomes because its magnitude is larger after easy than after difficult discriminations; it could be related to long-term changes in the decision process and could be useful for improving future decisions. These results are consistent with those interpretations that defend a differentiation in the functional significance of the positive and the negative performance-related ERP components (Overbeek et al., 2005; Hughes and Yeung, 2011; Philiastides et al., 2011). Furthermore, the results of the AUC ROC indicate that the EEG data can be analyzed trial-by-trial in relation with performance monitoring. This provides the experimenter with a powerful tool for complementing the traditional analyses based on the grand average and for providing a description of the information conveyed by the EEG activity during single trials. This has potential relevance for studying the normal function of performance monitoring and also for further understanding of the factors that in some diseases provoke deficits in performance monitoring. The capacity to learn from outcomes differs among people; however, in some diseases like schizophrenia patients have deficits in a variety of executive control processes (Cohen et al., 1999; Laurens et al., 2003; Lee and Park, 2005; Kerns et al., 2008; Kerns, 2009). Among them, they persist in their erroneous behavior repeatedly, unable to change their strategy, due to deficits in the prediction of error likelihood that result in disturbance in evaluating outcomes (Malenka et al., 1982; Krawitz et al., 2011). ROC analysis can be useful for studying deficits in using performance monitoring processes to shape future behavior; for example, by identifying those trials in which the outcomes are adequately encoded in the EEG, it would be possible to know whether these deficits are mainly related to difficulties in discriminating correct from incorrect trials or in using this information to modify future behavior.

### Conflict of interest statement

The authors declare that the research was conducted in the absence of any commercial or financial relationships that could be construed as a potential conflict of interest.
