# Supplementary material for: EEG activity represents the correctness of perceptual decisions trial-by-trial
Source: Front Behav Neurosci. 2014 Mar 28;8:105. doi: 10.3389/fnbeh.2014.00105 (PMC3975098; doi:10.3389/fnbeh.2014.00105)
Supplement: Supplementary file 1 [file DataSheet1.PDF]

Supplementary Material

EEG activity represents the correctness of perceptual decisions trial-by-trial.

Jose L. Pardo-Vazquez[1, 3], Isabel Padrón[1, 2], José Fernández-Rey[2] and Carlos Acuña[1]

[1] Departamento de Fisiología, Facultad de Medicina and Complejo Hospitalario Universitario (CHUS), Universidad de Santiago de Compostela, Spain.
[2] Departamento de Psicología Social, Básica y Metodología, Facultad de Psicología, Universidad de Santiago de Compostela, Spain.
[3] Present address, Champalimaud Centre for the Unknown, Lisboa, Portugal

*** Correspondence:** Jose L. Pardo-Vazquez, PhD. Circuit Dynamics & Computational Laboratory. Champalimaud Centre for the Unknown. Av. Brasília s/n (Doca de Pedrouços). 1400-038 Lisboa, Portugal. Telf. +351213032411.
jose.pardovazquez@neuro.fchampalimaud.org

1. Supplementary Figures and Tables

1.1. Suplementary Tables

Supplementary Table 1. Minima AUC ROC (PM-N) obtained in each scalp location averaged across the sample. Sig/Rec, number of participants with significant AUC ROC values with respect to the total number of participants.

Electrode	Sig/Rec	Mean	Median	SD	Electrode	Sig/Rec	Mean	Median	SD
Fp1	12/12	0.38	0.36	0.03	***C6***	12/12	0.35	0.34	0.05
Fpz	12/12	0.37	0.37	0.03	***T4***	5/5	0.39	0.40	0.04
Fp2	12/12	0.37	0.37	0.03	***T3L***	10/10	0.36	0.36	0.04
F3A	12/12	0.37	0.37	0.02	***TCP1***	12/12	0.36	0.37	0.05
F4A	12/12	0.36	0.35	0.04	***C3P***	12/12	0.37	0.38	0.04
F7	10/11	0.38	0.39	0.03	***CP1***	12/12	0.39	0.38	0.03
F5	12/12	0.37	0.38	0.03	***PZA***	11/12	0.39	0.39	0.04
F3	12/12	0.37	0.38	0.03	***CP2***	12/12	0.39	0.39	0.04
F1	12/12	0.38	0.38	0.03	***C4P***	12/12	0.38	0.38	0.04
FZ	12/12	0.38	0.38	0.04	***TCP2***	12/12	0.36	0.37	0.06
F2	12/12	0.37	0.37	0.04	***T4L***	12/12	0.36	0.36	0.07
F4	12/12	0.36	0.36	0.04	***T5***	11/11	0.31	0.32	0.08
F6	11/11	0.36	0.37	0.05	***P5***	11/11	0.34	0.35	0.07
F8	9/10	0.37	0.37	0.04	***P3***	12/12	0.37	0.39	0.06
C7A	11/12	0.38	0.38	0.03	***P1***	10/12	0.38	0.40	0.05
C5A	11/12	0.37	0.37	0.03	***PZ***	11/12	0.40	0.40	0.05
C3A	11/12	0.38	0.38	0.03	***P2***	10/12	0.39	0.41	0.04
C1A	10/12	0.39	0.39	0.03	***P4***	12/12	0.39	0.41	0.05
CZA	10/12	0.39	0.40	0.04	***P6***	12/12	0.36	0.39	0.08
C2A	11/12	0.38	0.38	0.04	***T6***	11/12	0.33	0.32	0.07
C4A	12/12	0.37	0.36	0.04	***CB1***	10/11	0.32	0.30	0.08
C6A	11/11	0.36	0.34	0.05	***P3P***	12/12	0.34	0.36	0.09
C8A	8/8	0.34	0.34	0.04	***P1P***	11/12	0.36	0.36	0.08
T3	6/6	0.39	0.39	0.03	***PZP***	12/12	0.39	0.42	0.07
C5	12/12	0.37	0.37	0.05	***P2P***	11/12	0.38	0.40	0.06
C3	11/12	0.37	0.37	0.02	***P4P***	11/12	0.35	0.38	0.08
C1	10/12	0.39	0.38	0.02	***CB2***	12/12	0.34	0.35	0.06
CZ	11/12	0.39	0.38	0.04	***O1***	10/11	0.35	0.33	0.06
C2	12/12	0.39	0.39	0.03	***OZ***	12/12	0.35	0.36	0.07
C4	12/12	0.36	0.35	0.04	***O2***	11/12	0.35	0.36	0.07

Supplementary Table 2. Maxima AUC ROC (PM-P) obtained in each scalp location averaged across the sample. Sig/Rec, number of participants with significant AUC ROC values with respect to the total number of participants.

Electrode	Sig/Rec	Mean	Median	SD	Electrode	Sig/Rec	Mean	Median	SD
Fp1	10/12	0.62	0.60	0.08	***C6***	11/12	0.69	0.71	0.04
Fpz	10/12	0.63	0.61	0.08	***T4***	5/5	0.63	0.64	0.06
Fp2	11/12	0.62	0.58	0.07	***T3L***	9/10	0.62	0.63	0.05
F3A	11/12	0.64	0.60	0.08	***TCP1***	12/12	0.66	0.66	0.06
F4A	12/12	0.64	0.62	0.08	***C3P***	12/12	0.69	0.70	0.06
F7	8/11	0.61	0.62	0.05	***CP1***	12/12	0.70	0.71	0.06
F5	11/12	0.64	0.60	0.08	***PZA***	12/12	0.71	0.72	0.07
F3	12/12	0.66	0.63	0.08	***CP2***	12/12	0.71	0.71	0.07
F1	12/12	0.69	0.67	0.09	***C4P***	12/12	0.70	0.72	0.07
FZ	12/12	0.71	0.68	0.09	***TCP2***	12/12	0.69	0.70	0.06
F2	12/12	0.71	0.67	0.08	***T4L***	11/12	0.65	0.64	0.05
F4	12/12	0.69	0.67	0.07	***T5***	10/11	0.65	0.65	0.05
F6	11/11	0.64	0.63	0.07	***P5***	11/11	0.67	0.66	0.06
F8	10/10	0.60	0.60	0.05	***P3***	12/12	0.69	0.69	0.05
C7A	8/12	0.63	0.63	0.04	***P1***	12/12	0.69	0.68	0.06
C5A	12/12	0.64	0.63	0.07	***PZ***	12/12	0.68	0.69	0.07
C3A	11/12	0.69	0.66	0.08	***P2***	12/12	0.68	0.69	0.07
C1A	11/12	0.73	0.70	0.07	***P4***	12/12	0.68	0.71	0.06
CZA	12/12	0.73	0.73	0.09	***P6***	12/12	0.68	0.67	0.06
C2A	11/12	0.74	0.73	0.07	***T6***	12/12	0.66	0.66	0.05
C4A	11/12	0.72	0.70	0.06	***CB1***	9/11	0.62	0.61	0.04
C6A	10/11	0.67	0.65	0.05	***P3P***	11/12	0.68	0.66	0.07
C8A	7/8	0.61	0.61	0.04	***P1P***	12/12	0.66	0.66	0.06
T3	3/6	0.61	0.62	0.01	***PZP***	12/12	0.65	0.66	0.06
C5	11/12	0.65	0.66	0.06	***P2P***	12/12	0.65	0.64	0.06
C3	11/12	0.70	0.70	0.06	***P4P***	12/12	0.64	0.63	0.06
C1	11/12	0.73	0.73	0.06	***CB2***	10/12	0.62	0.62	0.05
CZ	11/12	0.73	0.73	0.06	***O1***	11/11	0.62	0.61	0.05
C2	11/12	0.73	0.73	0.06	***OZ***	12/12	0.61	0.59	0.04
C4	11/12	0.72	0.72	0.05	***O2***	12/12	0.61	0.60	0.05

Supplementary Table 3. Latencies at minimum AUC ROC (PM-N) from feedback onset averaged across the sample.

Electrode	Mean	Median	SD	Electrode	Mean	Median	SD
Fp1	466	405	166	***C6***	266	342	149
Fpz	399	372	120	***T4***	276	334	258
Fp2	355	350	123	***T3L***	343	362	71
F3A	427	358	172	***TCP1***	269	323	127
F4A	327	350	157	***C3P***	257	321	138
F7	446	387	147	***CP1***	247	335	137
F5	403	357	159	***PZA***	235	334	146
F3	440	369	196	***CP2***	220	265	152
F1	366	356	133	***C4P***	207	291	171
FZ	294	347	152	***TCP2***	280	337	132
F2	303	346	142	***T4L***	343	342	142
F4	290	344	127	***T5***	289	316	90
F6	281	342	138	***P5***	275	308	91
F8	324	338	167	***P3***	276	333	128
C7A	295	340	92	***P1***	209	190	122
C5A	351	340	161	***PZ***	208	196	154
C3A	317	340	88	***P2***	255	338	135
C1A	261	327	128	***P4***	199	215	135
CZA	243	328	143	***P6***	220	264	132
C2A	235	336	160	***T6***	302	300	38
C4A	271	341	150	***CB1***	328	313	74
C6A	266	336	138	***P3P***	241	293	118
C8A	370	343	125	***P1P***	235	282	121
T3	294	363	130	***PZP***	247	307	125
C5	280	335	117	***P2P***	279	310	97
C3	295	338	103	***P4P***	261	308	99
C1	274	335	127	***CB2***	354	317	112
CZ	229	336	154	***O1***	330	314	154
C2	232	340	159	***OZ***	334	314	98
C4	273	346	148	***O2***	300	310	36

Supplementary Table 4. Latencies at maximum AUC ROC (PM-P) from feedback onset averaged across the sample.

Electrode	Mean	Median	SD	Electrode	Mean	Median	SD
Fp1	389	452	131	***C6***	498	476	58
Fpz	425	463	94	***T4***	503	490	37
Fp2	428	462	101	***T3L***	427	490	153
F3A	449	466	60	***TCP1***	450	489	107
F4A	463	462	21	***C3P***	443	480	101
F7	412	458	109	***CP1***	452	471	78
F5	451	464	63	***PZA***	453	471	78
F3	450	461	58	***CP2***	463	478	84
F1	462	461	21	***C4P***	458	470	84
FZ	461	461	20	***TCP2***	464	473	87
F2	463	460	23	***T4L***	499	482	54
F4	466	461	27	***T5***	448	512	173
F6	475	464	44	***P5***	446	496	130
F8	431	462	122	***P3***	416	470	124
C7A	437	456	132	***P1***	447	483	108
C5A	457	467	65	***PZ***	456	489	109
C3A	465	466	25	***P2***	467	484	89
C1A	464	466	23	***P4***	468	484	91
CZA	463	461	25	***P6***	463	486	137
C2A	468	464	28	***T6***	451	473	160
C4A	470	468	28	***CB1***	265	216	97
C6A	496	465	71	***P3P***	348	392	149
C8A	442	492	138	***P1P***	350	339	149
T3	481	580	171	***PZP***	398	432	145
C5	474	488	78	***P2P***	420	433	146
C3	474	474	31	***P4P***	367	409	152
C1	469	472	23	***CB2***	352	345	178
CZ	489	472	73	***O1***	289	216	109
C2	470	464	27	***OZ***	348	339	172
C4	478	470	35	***O2***	457	445	177

Supplementary Table 5. Results of *t*-tests comparing the magnitude of PM-N and PM-P.

	PM-N		PM-P			
Electrode	Mean	*SD*	Mean	*SD*	*t-value*	*p*
Fp1	0.12	0.03	0.12	0.08	-0.02	0.988
Fpz	0.12	0.03	0.13	0.08	-0.14	0.891
Fp2	0.13	0.03	0.12	0.07	0.64	0.539
F3A	0.13	0.03	0.14	0.08	-0.42	0.681
F4A	0.14	0.04	0.14	0.08	-0.08	0.938
F7	0.12	0.03	0.11	0.05	0.63	0.549
F5	0.13	0.03	0.14	0.08	-0.36	0.730
F3	0.12	0.03	0.13	0.08	-0.14	0.891
F1	**0.12**	**0.03**	**0.19**	**0.09**	**-2.51**	**0.029**
FZ	**0.12**	**0.04**	**0.21**	**0.09**	**-3.12**	**0.010**
F2	**0.13**	**0.04**	**0.21**	**0.08**	**-2.91**	**0.014**
F4	0.14	0.04	0.19	0.07	-1.94	0.078
F6	0.14	0.05	0.14	0.07	0.14	0.891
F8	0.13	0.04	0.10	0.05	1.28	0.238
C7A	0.12	0.04	0.13	0.04	-0.56	0.595
C5A	0.13	0.03	0.14	0.07	-0.48	0.642
C3A	**0.12**	**0.03**	**0.21**	**0.08**	**-2.60**	**0.029**
C1A	**0.11**	**0.03**	**0.24**	**0.08**	**-3.83**	**0.005**
CZA	**0.11**	**0.04**	**0.23**	**0.10**	**-3.00**	**0.015**
C2A	**0.11**	**0.04**	**0.24**	**0.07**	**-4.43**	**0.002**
C4A	**0.13**	**0.04**	**0.22**	**0.06**	**-4.23**	**0.002**
C6A	0.14	0.05	0.17	0.05	-1.30	0.227
C8A	**0.16**	**0.04**	**0.11**	**0.04**	**2.66**	**0.037**
T3	0.09	0.05	0.11	0.01	-0.63	0.593
C5	0.12	0.05	0.15	0.06	-1.51	0.162
C3	**0.13**	**0.02**	**0.20**	**0.06**	**-3.34**	**0.007**
C1	**0.11**	**0.03**	**0.23**	**0.07**	**-4.58**	**0.002**
CZ	**0.11**	**0.04**	**0.24**	**0.07**	**-5.11**	**0.001**
C2	**0.11**	**0.04**	**0.23**	**0.06**	**-5.57**	**0.000**
C4	**0.14**	**0.04**	**0.22**	**0.05**	**-4.88**	**0.001**
C6	**0.14**	**0.05**	**0.19**	**0.04**	**-3.21**	**0.009**
T4	0.11	0.04	0.13	0.06	-0.47	0.663
T3L	0.14	0.04	0.12	0.05	1.20	0.264
TCP1	0.14	0.05	0.16	0.06	-1.17	0.266
C3P	**0.13**	**0.04**	**0.19**	**0.06**	**-3.31**	**0.007**
CP1	**0.11**	**0.03**	**0.20**	**0.06**	**-4.79**	**0.001**
PZA	**0.11**	**0.04**	**0.20**	**0.07**	**-5.00**	**0.001**
CP2	**0.11**	**0.04**	**0.21**	**0.07**	**-4.63**	**0.001**
C4P	**0.12**	**0.04**	**0.20**	**0.07**	**-3.69**	**0.004**
TCP2	0.14	0.06	0.19	0.06	-1.76	0.106
T4L	0.15	0.07	0.15	0.05	-0.12	0.908
T5	**0.20**	**0.08**	**0.15**	**0.05**	**2.37**	**0.042**
P5	0.16	0.07	0.17	0.06	-0.48	0.641
P3	**0.13**	**0.06**	**0.19**	**0.05**	**-3.92**	**0.002**
P1	**0.12**	**0.05**	**0.17**	**0.06**	**-4.34**	**0.002**
PZ	**0.10**	**0.05**	**0.18**	**0.07**	**-4.07**	**0.002**
P2	**0.11**	**0.04**	**0.18**	**0.07**	**-3.29**	**0.009**
P4	**0.11**	**0.05**	**0.18**	**0.06**	**-2.95**	**0.013**
P6	0.14	0.08	0.18	0.06	-1.36	0.200
T6	0.17	0.07	0.16	0.05	0.32	0.754
CB1	**0.18**	**0.08**	**0.12**	**0.04**	**3.35**	**0.010**
P3P	0.16	0.09	0.18	0.07	-1.27	0.231
P1P	0.14	0.08	0.15	0.06	-0.67	0.519
PZP	0.11	0.07	0.15	0.06	-1.97	0.075
P2P	0.12	0.06	0.15	0.06	-1.31	0.221
P4P	0.15	0.08	0.15	0.06	0.08	0.934
CB2	0.15	0.06	0.12	0.05	1.64	0.136
O1	0.15	0.06	0.12	0.05	1.44	0.183
OZ	0.15	0.07	0.11	0.04	1.95	0.078
O2	0.15	0.07	0.11	0.05	2.00	0.073

Supplementary Table 6. Results of *t*-tests comparing the latency at PM-N and PM-P.

	PM-N		PM-P			
**Electrode**	**Mean**	***SD***	**Mean**	***SD***	***t-value***	***p***
Fp1	461	171	389	131	0,887	0,403
Fpz	377	106	425	94	-1,08	0,308
Fp2	329	92	428	101	-2,06	0,067
F3A	409	168	449	60	-0,67	0,516
F4A	**327**	**157**	**463**	**21**	**-2,87**	**0,015**
F7	461	172	399	111	0,87	0,416
F5	383	150	451	63	-1,26	0,236
F3	440	196	450	58	-0,16	0,875
F1	**366**	**133**	**462**	**21**	**-2,32**	**0,040**
FZ	**294**	**152**	**461**	**20**	**-3,74**	**0,003**
F2	**303**	**142**	**463**	**23**	**-3,80**	**0,003**
F4	**290**	**127**	**466**	**27**	**-4,57**	**0,001**
F6	**281**	**138**	**475**	**44**	**-4,43**	**0,001**
F8	324	167	425	128	-1,23	0,253
C7A	**283**	**100**	**459**	**125**	**-2,68**	**0,037**
C5A	**351**	**161**	**474**	**31**	**-2,48**	**0,033**
C3A	**310**	**90**	**468**	**24**	**-5,52**	**0,000**
C1A	**248**	**128**	**464**	**25**	**-4,99**	**0,001**
CZA	**243**	**143**	**466**	**26**	**-4,85**	**0,001**
C2A	**223**	**164**	**466**	**29**	**-4,71**	**0,001**
C4A	**263**	**155**	**470**	**28**	**-4,76**	**0,001**
C6A	**257**	**142**	**496**	**71**	**-5,92**	**0,000**
C8A	374	135	442	138	-0,77	0,469
T3	**212**	**146**	**481**	**171**	**-4,87**	**0,040**
C5	**269**	**117**	**474**	**78**	**-7,50**	**0,000**
C3	**285**	**103**	**477**	**31**	**-6,06**	**0,000**
C1	**262**	**129**	**471**	**24**	**-4,85**	**0,001**
CZ	**213**	**153**	**489**	**76**	**-5,92**	**0,000**
C2	**221**	**161**	**470**	**27**	**-5,10**	**0,000**
C4	**266**	**153**	**478**	**35**	**-4,96**	**0,001**
C6	**259**	**154**	**498**	**58**	**-5,71**	**0,000**
T4	276	258	503	37	-1,95	0,123
T3L	341	75	427	153	-1,57	0,156
TCP1	**269**	**127**	**450**	**107**	**-3,62**	**0,004**
C3P	**257**	**138**	**443**	**101**	**-3,49**	**0,005**
CP1	**247**	**137**	**452**	**78**	**-4,92**	**0,000**
PZA	**235**	**146**	**452**	**82**	**-4,60**	**0,001**
CP2	**220**	**152**	**463**	**84**	**-5,38**	**0,000**
C4P	**207**	**171**	**458**	**84**	**-4,30**	**0,001**
TCP2	**280**	**132**	**464**	**87**	**-4,13**	**0,002**
T4L	**343**	**149**	**499**	**54**	**-3,23**	**0,009**
T5	312	53	448	173	-2,24	0,052
P5	**275**	**91**	**446**	**130**	**-3,11**	**0,011**
P3	**276**	**128**	**416**	**124**	**-2,57**	**0,026**
P1	**209**	**122**	**466**	**99**	**-6,60**	**0,000**
PZ	**208**	**154**	**475**	**92**	**-5,72**	**0,000**
P2	**255**	**135**	**473**	**97**	**-4,81**	**0,001**
P4	**199**	**135**	**468**	**91**	**-6,23**	**0,000**
P6	**220**	**132**	**463**	**137**	**-4,36**	**0,001**
T6	**302**	**38**	**466**	**159**	**-3,91**	**0,003**
CB1	329	79	265	97	1,57	0,156
P3P	257	109	348	149	-1,78	0,105
P1P	235	121	359	153	-2,05	0,067
PZP	**247**	**125**	**398**	**145**	**-2,52**	**0,028**
P2P	**279**	**97**	**432**	**146**	**-2,55**	**0,029**
P4P	261	99	374	157	-1,68	0,123
CB2	349	119	352	178	-0,05	0,963
O1	330	154	290	115	0,66	0,526
OZ	334	98	348	172	-0,25	0,810
O2	**300**	**36**	**473**	**177**	**-2,98**	**0,014**

Supplementary Table 7. Results of t-tests comparing the minima values reached by PM-N after easy and difficult trials.

	Easy		Difficult					Easy		Difficult			
**Electrode**	**Mean**	**SD**	**Mean**	**SD**	***t-value***	***p***	**Electrode**	**Mean**	**SD**	**Mean**	**SD**	***t-value***	***p***
Fp1	0.34	0.06	0.36	0.03	-1.22	0.24	***C6***	0.34	0.05	0.32	0.06	0.91	0.38
Fpz	0.34	0.05	0.35	0.03	-0.47	0.65	***T4***	0.35	0.06	0.38	0.04	-1.03	0.34
Fp2	0.35	0.06	0.34	0.03	0.52	0.61	***T3L***	0.34	0.05	0.34	0.03	0.05	0.96
F3A	0.35	0.05	0.35	0.04	0.39	0.70	***TCP1***	0.35	0.04	0.34	0.04	0.19	0.85
F4A	0.37	0.03	0.32	0.06	1.83	0.09	***C3P***	0.35	0.04	0.35	0.05	-0.16	0.87
F7	0.35	0.05	0.38	0.05	-1.47	0.16	***CP1***	0.36	0.05	0.37	0.06	-0.31	0.76
F5	0.35	0.03	0.35	0.04	-0.34	0.74	***PZA***	0.35	0.03	0.36	0.04	-0.66	0.52
F3	0.35	0.03	0.34	0.04	0.59	0.56	***CP2***	0.35	0.03	0.36	0.05	-0.48	0.64
F1	0.35	0.05	0.34	0.04	0.38	0.71	***C4P***	0.35	0.04	0.35	0.07	-0.07	0.94
FZ	0.35	0.04	0.34	0.04	0.61	0.55	***TCP2***	0.35	0.04	0.33	0.05	0.76	0.46
F2	0.36	0.04	0.34	0.05	1.12	0.28	***T4L***	0.35	0.05	0.35	0.04	-0.25	0.81
F4	0.35	0.03	0.32	0.06	1.22	0.24	***T5***	0.31	0.08	0.31	0.04	0.20	0.84
F6	0.36	0.03	0.34	0.05	1.08	0.30	***P5***	0.33	0.05	0.34	0.04	-0.09	0.93
F8	0.35	0.04	0.35	0.05	-0.07	0.95	***P3***	0.35	0.03	0.35	0.04	0.07	0.95
C7A	0.35	0.03	0.38	0.05	-1.34	0.20	***P1***	0.38	0.03	0.37	0.04	0.48	0.64
C5A	0.35	0.05	0.36	0.06	-0.59	0.57	***PZ***	0.39	0.02	0.40	0.06	-0.14	0.89
C3A	0.35	0.05	0.35	0.04	0.02	0.99	***P2***	0.36	0.03	0.38	0.06	-0.50	0.63
C1A	0.35	0.05	0.36	0.04	-0.18	0.86	***P4***	0.32	0.04	0.35	0.04	-1.14	0.28
CZA	0.35	0.04	0.36	0.05	-0.33	0.75	***P6***	0.33	0.07	0.34	0.05	-0.41	0.69
C2A	0.34	0.04	0.34	0.05	-0.11	0.92	***T6***	0.33	0.05	0.33	0.04	0.15	0.88
C4A	0.33	0.04	0.32	0.06	0.40	0.69	***CB1***	0.30	0.09	0.34	0.07	-1.18	0.26
C6A	0.34	0.04	0.33	0.05	0.33	0.75	***P3P***	0.30	0.07	0.35	0.04	-1.68	0.12
C8A	0.33	0.04	0.33	0.04	-0.10	0.92	***P1P***	0.33	0.05	0.36	0.04	-1.22	0.24
T3	0.33	0.05	0.38	0.04	-2.14	0.06	***PZP***	0.36	0.03	0.38	0.04	-0.64	0.53
C5	0.35	0.04	0.35	0.06	-0.22	0.83	***P2P***	0.35	0.03	0.37	0.04	-1.17	0.27
C3	0.35	0.05	0.35	0.06	-0.01	0.99	***P4P***	0.32	0.06	0.35	0.04	-1.12	0.29
C1	0.33	0.04	0.36	0.04	-0.98	0.34	***CB2***	0.33	0.04	0.35	0.04	-1.49	0.16
CZ	0.35	0.03	0.36	0.05	-0.62	0.54	***O1***	0.31	0.07	0.35	0.07	-1.28	0.22
C2	0.34	0.04	0.34	0.05	-0.32	0.75	***OZ***	0.33	0.07	0.36	0.03	-1.04	0.32
C4	0.33	0.04	0.33	0.07	0.08	0.93	***O2***	0.34	0.05	0.36	0.04	-0.92	0.37

Supplementary Table 8. Results of *t*-tests comparing the latency of PM-N after easy and difficult trials.

	Easy		Difficult					Easy		Difficult			
**Electrode**	**Mean**	**SD**	**Mean**	**SD**	***t-value***	***p***	**Electrode**	**Mean**	**SD**	**Mean**	**SD**	***t-value***	***p***
Fp1	390	220	422	119	-0.37	0.72	***C6***	**108**	**133**	**349**	**18**	**-5.41**	**0.00**
Fpz	402	158	386	92	0.25	0.81	***T4***	93	161	286	159	-1.81	0.11
Fp2	**226**	**168**	**387**	**93**	**-2.47**	**0.03**	***T3L***	**243**	**137**	**367**	**18**	**-2.55**	**0.02**
F3A	368	247	385	92	-0.20	0.85	***TCP1***	**173**	**150**	**346**	**44**	**-3.31**	**0.00**
F4A	**190**	**218**	**385**	**93**	**-2.43**	**0.03**	***C3P***	**62**	**65**	**316**	**79**	**-7.21**	**0.00**
F7	424	240	440	160	-0.15	0.88	***CP1***	**38**	**40**	**300**	**87**	**-7.76**	**0.00**
F5	342	284	430	158	-0.78	0.45	***PZA***	**31**	**31**	**298**	**79**	**-8.35**	**0.00**
F3	232	265	386	90	-1.64	0.12	***CP2***	**36**	**40**	**316**	**100**	**-6.93**	**0.00**
F1	**50**	**32**	**385**	**93**	**-8.43**	**0.00**	***C4P***	**64**	**79**	**350**	**14**	**-10.09**	**0.00**
FZ	**119**	**186**	**336**	**53**	**-3.36**	**0.00**	***TCP2***	**105**	**115**	**339**	**33**	**-5.85**	**0.00**
F2	**110**	**174**	**382**	**94**	**-4.09**	**0.00**	***T4L***	**139**	**140**	**367**	**114**	**-3.70**	**0.00**
F4	**67**	**68**	**349**	**16**	**-12.02**	**0.00**	***T5***	241	147	313	58	-1.30	0.22
F6	**100**	**90**	**343**	**20**	**-7.49**	**0.00**	***P5***	327	259	317	58	0.11	0.91
F8	**180**	**139**	**348**	**25**	**-3.39**	**0.00**	***P3***	**91**	**128**	**369**	**130**	**-4.08**	**0.00**
C7A	285	257	365	23	-0.89	0.39	***P1***	**95**	**125**	**296**	**79**	**-3.73**	**0.00**
C5A	279	317	388	94	-0.93	0.37	***PZ***	**81**	**51**	**300**	**75**	**-4.54**	**0.00**
C3A	**70**	**37**	**338**	**55**	**-11.11**	**0.00**	***P2***	231	234	314	60	-1.00	0.34
C1A	**49**	**27**	**335**	**54**	**-12.82**	**0.00**	***P4***	**130**	**82**	**304**	**61**	**-3.99**	**0.00**
CZA	**43**	**29**	**330**	**54**	**-12.70**	**0.00**	***P6***	**141**	**128**	**299**	**53**	**-3.32**	**0.01**
C2A	**41**	**27**	**334**	**54**	**-13.16**	**0.00**	***T6***	312	184	310	40	0.02	0.98
C4A	**41**	**31**	**348**	**18**	**-25.41**	**0.00**	***CB1***	362	178	319	128	0.55	0.59
C6A	**133**	**126**	**350**	**22**	**-4.78**	**0.00**	***P3P***	412	183	305	50	1.69	0.12
C8A	**154**	**98**	**352**	**25**	**-4.80**	**0.00**	***P1P***	412	184	363	130	0.59	0.57
T3	**213**	**139**	**359**	**61**	**-2.36**	**0.04**	***PZP***	**468**	**204**	**303**	**58**	**2.33**	**0.04**
C5	209	262	361	19	-1.65	0.12	***P2P***	254	197	292	58	-0.57	0.58
C3	**56**	**22**	**333**	**57**	**-12.75**	**0.00**	***P4P***	326	270	281	54	0.49	0.63
C1	**48**	**28**	**336**	**54**	**-11.98**	**0.00**	***CB2***	387	169	344	107	0.63	0.54
CZ	**36**	**31**	**335**	**54**	**-13.04**	**0.00**	***O1***	392	167	298	49	1.53	0.15
C2	**32**	**30**	**331**	**54**	**-13.98**	**0.00**	***OZ***	407	179	303	48	1.68	0.12
C4	**38**	**35**	**351**	**18**	**-22.27**	**0.00**	***O2***	397	171	284	51	1.89	0.08

Supplementary Table 9. Results of t-tests comparing the maxima values reached by PM-P after easy and difficult trials.

	Easy		Difficult					Easy		Difficult			
Electrode	Mean	SD	Mean	SD	*t-value*	*p*	Electrode	Mean	SD	Mean	SD	*t-value*	*p*
Fp1	0.70	0.10	0.62	0.06	1.77	0.11	*C6*	**0.77**	**0.06**	**0.65**	**0.06**	**3.96**	**0.00**
Fpz	0.68	0.09	0.63	0.06	1.32	0.22	*T4*	0.73	0.04	0.63	0.06	2.35	0.07
Fp2	0.66	0.09	0.64	0.08	0.31	0.76	*T3L*	**0.67**	**0.06**	**0.61**	**0.03**	**2.26**	**0.05**
F3A	0.71	0.10	0.63	0.07	1.66	0.13	*TCP1*	**0.72**	**0.05**	**0.63**	**0.03**	**4.33**	**0.00**
F4A	0.69	0.08	0.65	0.08	0.94	0.37	*C3P*	**0.77**	**0.05**	**0.65**	**0.04**	**5.22**	**0.00**
F7	0.68	0.08	0.61	0.04	1.93	0.09	*CP1*	**0.79**	**0.06**	**0.66**	**0.05**	**5.27**	**0.00**
F5	**0.71**	**0.09**	**0.62**	**0.06**	**2.20**	**0.05**	*PZA*	**0.80**	**0.07**	**0.67**	**0.05**	**4.22**	**0.00**
F3	0.71	0.10	0.64	0.07	1.65	0.12	*CP2*	**0.77**	**0.10**	**0.67**	**0.06**	**2.55**	**0.02**
F1	0.72	0.09	0.67	0.07	1.18	0.26	*C4P*	**0.77**	**0.10**	**0.67**	**0.05**	**2.66**	**0.02**
FZ	0.73	0.09	0.68	0.08	1.14	0.27	*TCP2*	**0.76**	**0.09**	**0.66**	**0.05**	**2.85**	**0.01**
F2	0.73	0.08	0.69	0.07	1.08	0.30	*T4L*	**0.73**	**0.08**	**0.63**	**0.05**	**3.02**	**0.01**
F4	0.72	0.08	0.67	0.07	1.21	0.25	*T5*	0.70	0.06	0.65	0.05	1.53	0.15
F6	0.69	0.08	0.63	0.07	1.45	0.18	*P5*	0.73	0.05	0.69	0.06	1.55	0.15
F8	0.67	0.04	0.62	0.04	1.87	0.10	*P3*	**0.75**	**0.05**	**0.66**	**0.05**	**3.57**	**0.00**
C7A	0.68	0.07	0.62	0.03	2.10	0.07	*P1*	**0.76**	**0.07**	**0.66**	**0.04**	**3.64**	**0.00**
C5A	0.70	0.09	0.64	0.06	1.24	0.24	*PZ*	**0.76**	**0.08**	**0.65**	**0.04**	**3.38**	**0.00**
C3A	0.72	0.09	0.65	0.07	1.89	0.08	*P2*	**0.75**	**0.08**	**0.65**	**0.03**	**2.98**	**0.01**
C1A	0.75	0.08	0.69	0.07	1.90	0.08	*P4*	**0.75**	**0.07**	**0.65**	**0.05**	**3.26**	**0.00**
CZA	0.77	0.08	0.72	0.07	1.34	0.20	*P6*	**0.74**	**0.08**	**0.65**	**0.05**	**2.91**	**0.01**
C2A	**0.78**	**0.07**	**0.71**	**0.06**	**2.15**	**0.05**	*T6*	**0.72**	**0.07**	**0.65**	**0.05**	**2.45**	**0.03**
C4A	**0.77**	**0.05**	**0.69**	**0.05**	**3.29**	**0.01**	*CB1*	0.66	0.06	0.63	0.04	1.11	0.29
C6A	**0.73**	**0.06**	**0.65**	**0.06**	**2.60**	**0.02**	*P3P*	**0.74**	**0.05**	**0.65**	**0.07**	**2.94**	**0.01**
C8A	**0.69**	**0.06**	**0.61**	**0.04**	**2.37**	**0.05**	*P1P*	**0.72**	**0.04**	**0.65**	**0.06**	**3.14**	**0.01**
T3	0.64	0.04	0.62	0.02	0.89	0.42	*PZP*	**0.72**	**0.07**	**0.63**	**0.04**	**3.04**	**0.01**
C5	**0.70**	**0.07**	**0.63**	**0.04**	**2.57**	**0.02**	*P2P*	**0.70**	**0.07**	**0.63**	**0.03**	**2.60**	**0.02**
C3	**0.75**	**0.07**	**0.65**	**0.06**	**3.25**	**0.01**	*P4P*	0.69	0.07	0.64	0.04	1.76	0.10
C1	**0.77**	**0.06**	**0.67**	**0.06**	**3.21**	**0.01**	*CB2*	0.66	0.06	0.63	0.04	1.19	0.26
CZ	**0.78**	**0.07**	**0.69**	**0.07**	**2.81**	**0.01**	*O1*	0.69	0.05	0.65	0.05	1.66	0.12
C2	**0.80**	**0.07**	**0.69**	**0.07**	**3.39**	**0.00**	*OZ*	0.65	0.06	0.63	0.04	0.95	0.36
C4	**0.79**	**0.06**	**0.68**	**0.04**	**4.14**	**0.00**	*O2*	0.66	0.06	0.63	0.01	1.14	0.28

Supplementary Table 10. Results of *t*-tests comparing the latency of PM-P after easy and difficult trials.

	Easy		Difficult					Easy		Difficult			
**Electrode**	**Mean**	**SD**	**Mean**	**SD**	***t-value***	***p***	**Electrode**	**Mean**	**SD**	**Mean**	**SD**	***t-value***	***p***
Fp1	379	153	350	179	0.27	0.79	***C6***	481	39	500	44	-0.95	0.36
Fpz	486	59	383	162	1.33	0.21	***T4***	475	30	448	158	0.29	0.78
Fp2	393	159	372	130	0.23	0.82	***T3L***	433	120	373	190	0.66	0.52
F3A	486	61	396	143	1.33	0.21	***TCP1***	418	97	437	154	-0.31	0.76
F4A	434	85	464	17	-0.92	0.37	***C3P***	417	96	439	92	-0.47	0.65
F7	396	118	369	136	0.33	0.75	***CP1***	442	78	444	86	-0.03	0.97
F5	415	109	368	153	0.59	0.56	***PZA***	445	78	504	77	-1.55	0.14
F3	419	97	465	15	-1.43	0.18	***CP2***	464	18	468	121	-0.11	0.91
F1	437	64	464	18	-1.21	0.24	***C4P***	458	24	447	89	0.36	0.72
FZ	449	21	463	17	-1.48	0.16	***TCP2***	472	32	458	95	0.42	0.68
F2	453	16	462	15	-1.16	0.27	***T4L***	499	88	498	124	0.03	0.98
F4	461	19	462	18	-0.11	0.92	***T5***	380	144	393	179	-0.16	0.88
F6	448	13	478	48	-1.47	0.17	***P5***	311	127	402	160	-1.19	0.26
F8	402	100	494	60	-1.76	0.12	***P3***	340	116	393	150	-0.82	0.42
C7A	430	60	347	145	1.06	0.32	***P1***	412	102	381	135	0.55	0.59
C5A	484	52	346	143	2.22	0.05	***PZ***	380	120	438	137	-0.92	0.37
C3A	437	70	467	20	-1.25	0.23	***P2***	380	120	436	93	-1.05	0.31
C1A	460	18	465	18	-0.66	0.52	***P4***	434	84	465	102	-0.71	0.49
CZA	455	20	492	89	-1.14	0.27	***P6***	436	92	431	136	0.10	0.92
C2A	458	20	494	87	-1.14	0.27	***T6***	429	102	307	167	1.87	0.08
C4A	459	20	469	20	-0.95	0.36	***CB1***	324	201	298	108	0.33	0.75
C6A	452	58	480	45	-1.02	0.33	***P3P***	339	119	357	167	-0.26	0.80
C8A	438	114	451	131	-0.16	0.88	***P1P***	288	127	367	154	-1.15	0.27
T3	401	124	359	197	0.31	0.77	***PZP***	354	145	466	159	-1.52	0.15
C5	423	99	457	88	-0.71	0.49	***P2P***	337	135	454	161	-1.64	0.12
C3	443	72	470	21	-0.99	0.34	***P4P***	331	148	443	123	-1.62	0.13
C1	468	22	497	83	-0.97	0.35	***CB2***	337	173	261	103	1.01	0.33
CZ	462	23	498	80	-1.23	0.24	***O1***	356	153	325	119	0.44	0.67
C2	462	21	498	78	-1.24	0.23	***OZ***	419	163	363	203	0.59	0.57
C4	462	23	471	17	-0.84	0.42	***O2***	411	152	489	96	-1.09	0.30

1.2. Suplementary Figures

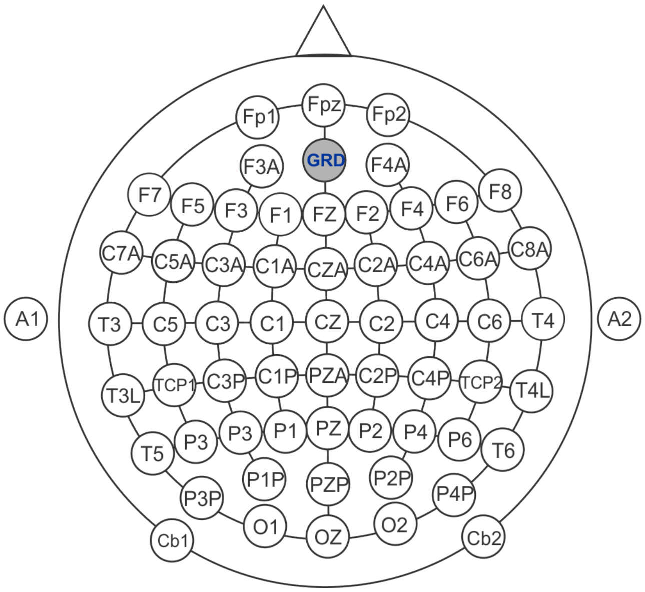

Supplementary Figure 1. Electrode localizations. Schematic representation of the electrode localizations.

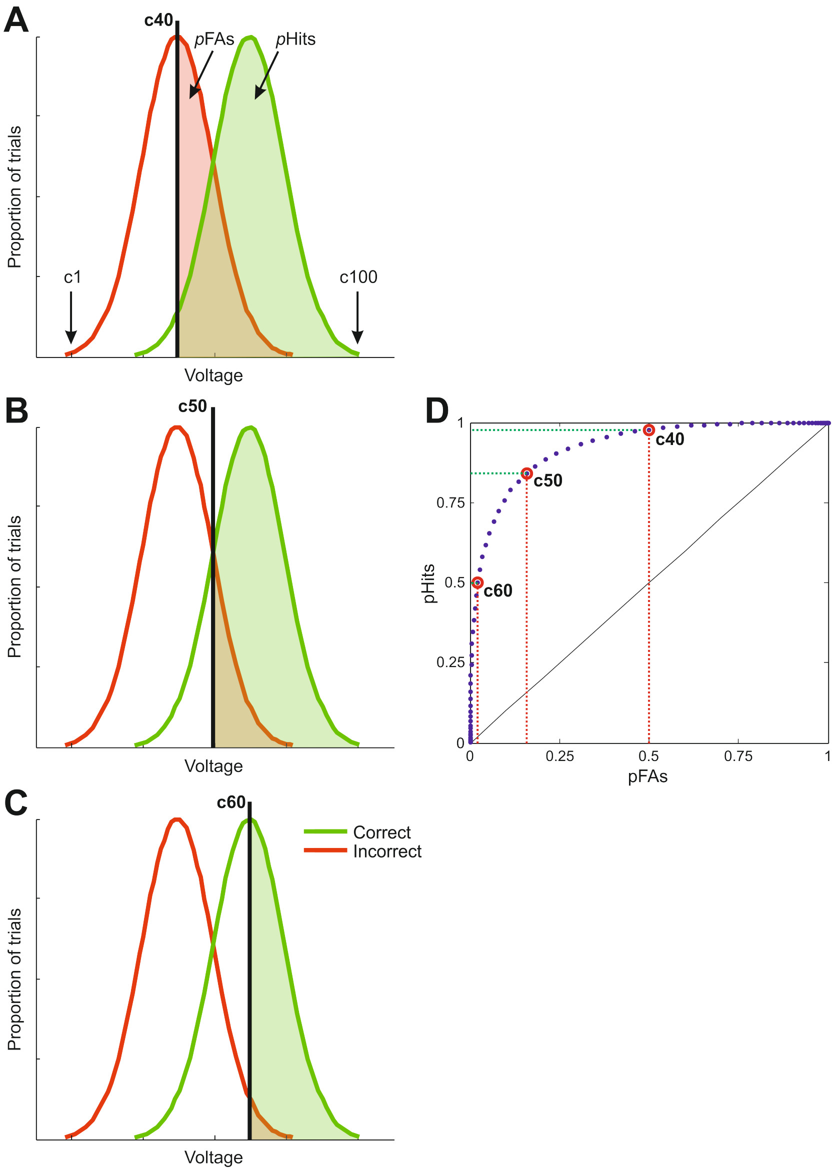

Supplementary Figure 2. Explanation of the procedure used to estimate the ROC curve. (A) Distributions of (simulated) voltages, for correct and incorrect trials, at a given time. To estimate the proportion of hits (*p*Hits) and false alarms (*p*FAs), the distributions of voltages are compared with a criterion (in this case c40). *p*Hits (green shaded area) is defined as the proportion of correct trials in which the voltage is equal to or higher than the criterion. Conversely, *p*FAs (red shaded area) is defined as the proportion of incorrect trials in which the voltage was equal to or higher than the criterion. **(B, C)** Proportion of hits and false alarms using two different criteria (c50 and c60). **(D)** ROC curve corresponding to the distributions depicted in A, B and C. The ROC curve represents the *p*Hits against *p*FAs for a set of 100 criteria, from the minimum to the maximum voltages (c1 and c100, respectively, in panel A). The three points corresponding to the criteria used in A, B and C are indicated with red halos and labels (c40, c50 and c60, respectively).

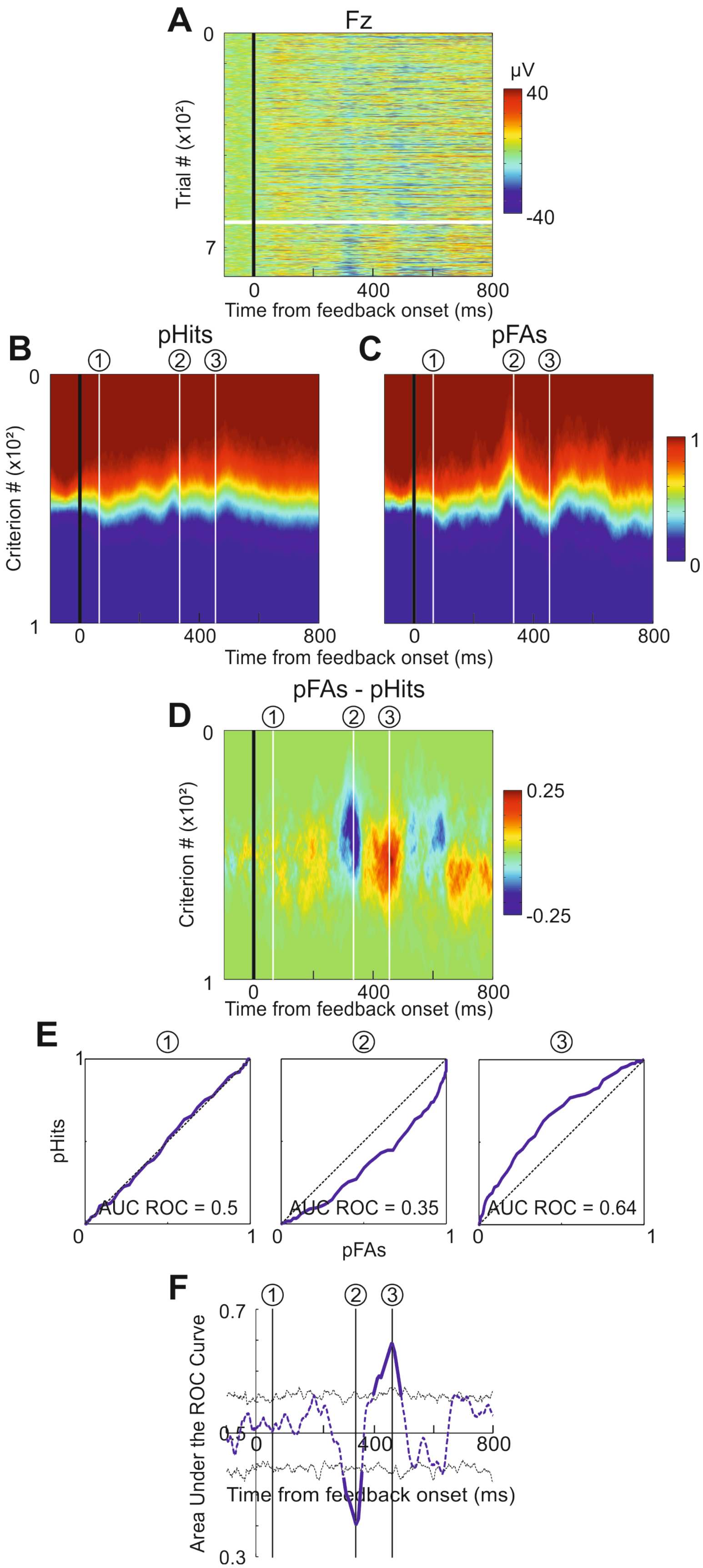
A
Fz
0
7
Trial # (x10²)
μV
40
-40
0
400
800
Time from feedback onset (ms)
B
pHits
C
pFAs
①
②
③
Criterion # (x10²)
1
0
Time from feedback onset (ms)
D
pFAs - pHits
0.25
-0.25
E
pHits
AUC ROC = 0.5
AUC ROC = 0.35
AUC ROC = 0.64
pFAs
F
0.7
0.5
0.3
Area Under the ROC Curve
Time from feedback onset (ms)

Supplementary Figure 3. Trial-by-trial analysis confirms the existence of two ERP components that differentiate correct from incorrect trials after feedback presentation. (A) Voltage, as a function of time, recorded in one electrode located at FZ for one example participant; each line represents a trial and the color code, from blue to red, the voltage; vertical black line indicates the presentation of the feedback; horizontal white line separates correct (upper) from incorrect (lower) trials. **(B, C)** Proportion of correct (pHits) and incorrect (pFAs) trials, respectively, that surpasses a criterion, as a function of time and criterion. **(D)** Difference between pFAs and pHits as a function of time and criterion. **(E)** Area under the ROC curve (AUC ROC) estimated at different time points [(1), (2) and (3)] that are signaled in B, C and D with vertical white lines. **(F)** AUC ROC as a function of time (blue line); continuous traces indicate significant periods; black upper and lower dashed lines represent the significance thresholds (estimated using a permutation test, n=200 iterations); vertical black lines, labeled with (1), (2) and (3) indicate the time points corresponding to instant AUC ROCs represented in E.

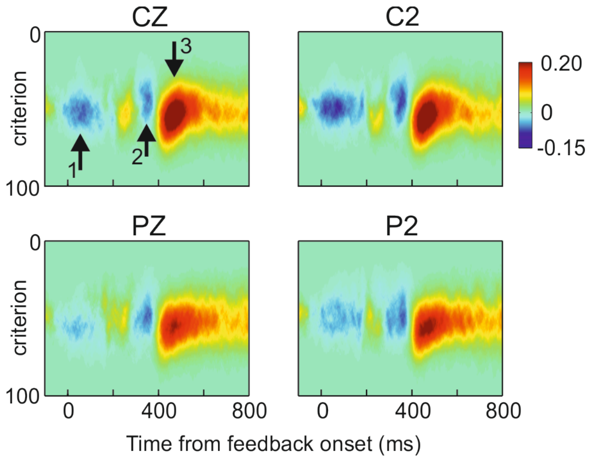

Supplementary Figure 4. Temporal evolution of the differences between *p*FAs and *p*Hits. Difference between *p*FAs and *p*Hits averaged across the sample (N=12), as a function of time and criterion, estimated at different electrode positions. Arrows 1 and 2 signal the two negative and arrow 3 the positive relevant periods.

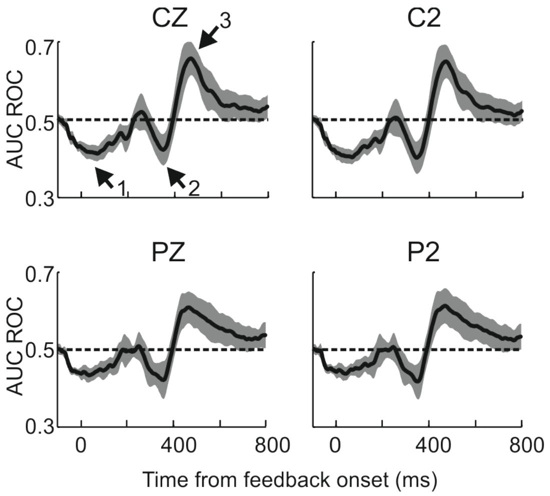

Supplementary Figure 5. Temporal evolution of the area under the ROC curve. AUC ROC (*Mean*±2*SEM*), comparing the voltage recorded at different electrodes after correct and incorrect trials, averaged across the sample (N=12), as a function of time. Arrows signal the two negative (1 and 2) and the positive (3) components.

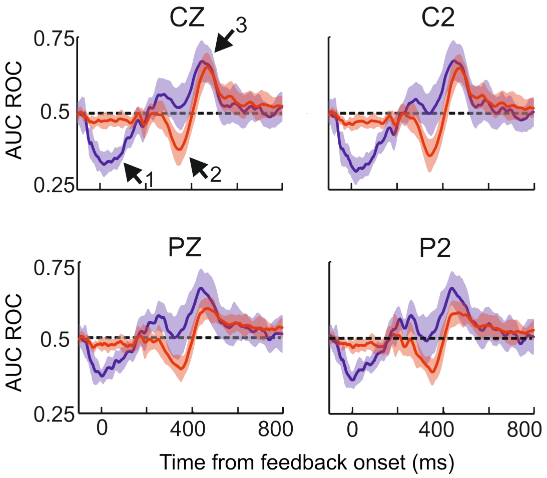

Supplementary Figure 6. Temporal evolution of the AUC ROC as a function of the task difficulty. Area under the ROC curve (Mean±2SEM), comparing the voltage recorded at different electrodes after correct and incorrect trials, averaged across the sample (N=9) as a function of time and difficulty of the discriminations; blue and red lines represent the AUC ROC for easy and difficult discriminations, respectively. Arrows signal the relevant components: 1, negative component for the easy trials only; 2, negative component present only after difficult trials; and 3, positive component after both easy and difficult trials.

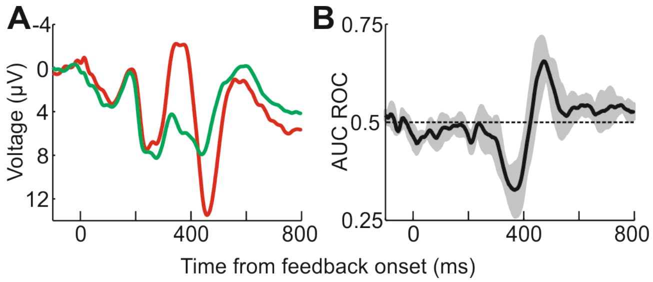

Supplementary Figure 7. The difference between the frequencies of correct and incorrect trials does not explain the PM-N and PM-P components. (A) mean voltage averaged across the sample (5 participants) recorded at FZ, in a control experiment in which the frequencies of correct and incorrect trials were equaled. Green and red lines for correct and incorrect trials, respectively. **(B)** Area under the ROC curve (Mean±2SEM), comparing the voltage recorded at Fz after correct and incorrect trials, averaged across the sample (5 participants) as a function of time.
